# Synthesis of the Fatty Esters of Solketal and Glycerol-Formal: Biobased Specialty Chemicals

**DOI:** 10.3390/molecules21020170

**Published:** 2016-01-30

**Authors:** Alvise Perosa, Andrea Moraschini, Maurizio Selva, Marco Noè

**Affiliations:** Dipartimento di Scienze Molecolari e Nanosistemi, Centro per le Tecnologie Chimiche Sostenibili, Università Ca’ Foscari Venezia, Via Torino 155, Venezia Mestre 30172, Italy; moraschini@libero.it (A.M.); selva@unive.it (M.S.); marco.noe@unive.it (M.N.)

**Keywords:** glycerol, solketal, glycerol formal, fatty esters, bio-based molecules

## Abstract

The caprylic, lauric, palmitic and stearic esters of solketal and glycerol formal were synthesized with high selectivity and in good yields by a solvent-free acid catalyzed procedure. No acetal hydrolysis was observed, notwithstanding the acidic reaction conditions.

## 1. Introduction

The impact on the environment of fossil-based fuels and chemicals is a matter of widespread concern. It is therefore desirable—if not unavoidable—to investigate new feedstocks, products and processes with lower environmental impact and that are sustainable in the longer term [1]. For example, in the recent past the growing production and use of carbon-neutral biofuels such as biodiesel has become an option and a valuable addition to conventional fuels [2], as justified by two basic needs of the economic model in the industrialised countries. First, the need to reduce the dependence on imported oil both in terms of price stability as well as in terms of political stability. Second, the need to reduce emissions associated with fossil-based diesel combustion in engines, with special emphasis in CO, CO_2_, SO_X_, C and particulate matter, which decrease using biodiesel [3]. Currently, most biodiesel derives from the transesterification reaction between an oil and an alcohol such as methanol. This reaction provides two main products: the methyl esters of the fatty acids, *i.e.*, biodiesel itself, and an important by-product: glycerol. Crude glycerol is nowadays the most abundant renewable chemical feedstock in the world [4] and as such, it is a promising starting material to produce higher value-added chemicals. In countries with high volumes of biodiesel production, this by-product may even represent a disposal problem [5]. Prior to chemical upgrading, crude glycerol normally requires a first purification step [6]. The subsequent steps, e.g., upgrading to biofuels and fuel components, are widely investigated processes [7]. For example, recent publications have described different synthetic pathways to obtain a variety of glycerol derivatives by microbial fermentation [8], ketalization [9], acetylation [10], etherification [11], transesterification [12], catalytic decomposition [13], or combinations thereof [14]. In particular, acid-catalyzed condensation of glycerol with acetone [15,16], acetaldehyde or formaldehyde (or dimethoxymethane) yields the products solketal^®^, glycerol acetal and glycerol formal (GlyF), which are interesting because they can be used directly as fuel additives [17,18] that reduce soot formation [19,20] or as reagents for further production of biofuel components [21]. GlyF—the acetal of glycerol with formaldehyde—exists as a mixture of two isomers (5- and 6-membered rings in a 2:3 molar ratio, respectively), while solketal—the ketal of glycerol with acetone—is present as a single five-membered ring product [22,23,24,25]. Ketals are considered a subclass of acetals and we will therefore refer to both as acetals. Both these acetals are currently used mainly as solvents for injectable pharmaceutical preparations, paints and waterbased inks, and for the preparation of additives for biodiesel formulations [10,25,26,27,28,29,30,31,32,33]. However, GlyF and solketal are also valuable glycerol synthons due to the presence of a free hydroxyl functionality available for esterification and etherification reactions.

**Scheme 1:**
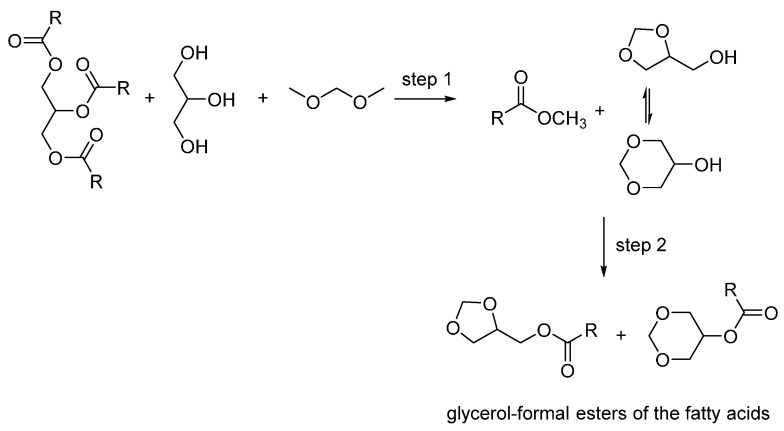
Process for the synthesis of the fatty acid esters of glycerol-formal [7].

Recently, a combined transesterification-acetalization approach towards the synthesis of a class of potential fuel additives based on crude glycerol and waste oils was described [7]. Specifically, a process was proposed whereby starting from a feed constituted by waste triglycerides, glycerol and dimethoxymethane as acetalization reagent, the glycerol-formal esters of the fatty acids were obtained, either in pure form or as a blend with the methyl esters of the fatty acids (biodiesel) (Scheme 1). The physical properties of the pure glycerol-formal esters of the fatty acids or of the blend containing the methyl esters were analyzed as diesel additives. These compounds have higher lubricity and higher cetane numbers compared to pure biodiesel. Nonetheless, the results indicated that this biofuel or its blends with diesel fuels require additional studies.

The work described in the present paper originates from the idea of combining glycerol with free fatty acids (FFAs) for the synthesis of new totally biobased chemicals. FFAs are established platform chemicals derived from triglycerides by saponification and as such represent oleochemicals of renewable origin obtainable from biorefinery process streams. In fact, soaps were the predominant products obtained from vegetable oils before the advent of biodiesel. Several FFAs are still valuable oleochemicals used for a variety of applications [34], implying that the technologies for their production are well established and that the renewed large availability of vegetable oils may provide a driver for their use as building blocks for new bio-based chemicals. In particular, we here describe the direct acid-catalyzed synthesis and characterization of fatty acids solketal esters (FASEs) and fatty acid glycerol formal esters (FAGEs).

## 2. Results and Discussion 

The target molecules were synthesized as shown in Equations (1) and (2), by acid catalyzed esterification of four different FFAs: caprylic (CH_3_(CH_2_)_6_COOH, C8:0), lauric (CH_3_(CH_2_)_10_COOH, C12:0), palmitic (CH_3_(CH_2_)_14_COOH, C16:0) and stearic (CH_3_(CH_2_)_16_COOH, C18:0) with the glycerol acetals solketal and GlyF. Glycerol was incorporated in the products in the form of its acetals—solketal and glycerol formal (GlyF)—two compounds that are already used as constituents of additives for biodiesel formulations [26,27,28,29,30,31]. Both possess one free hydroxyl group available for functionalization. Generally, one of the main issues associated with using these acetals is their reported lack of stability under acidic conditions [35]. Here we demonstrate that it is possible to carry out the esterification of solketal and GlyF with free fatty acids under acid catalysis with no concurrent acetal ring-opening, under mild operative conditions and in the presence of low concentrations of *p*-toleuensulfonic acid (PTSA).

**Figure 1:**
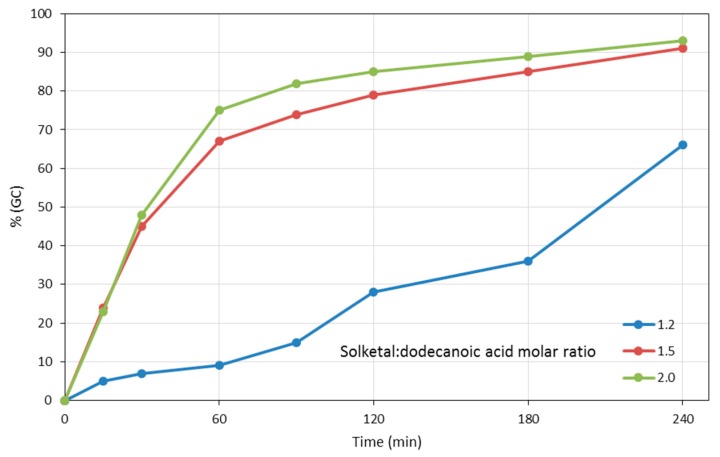
Time-dependent formation of (2,2-dimethyl-1,3-dioxolan-4-yl)methyl dodecanoate (**2**) at T = 60 °C in the presence of 5% PTSA with different solketal-dodecanoic acid molar ratios.

A preliminary screening of catalyst type (H_2_SO_4_ *vs.* PTSA) and its concentration (5, 10%), of the effect of added solvents (acetone), of reaction temperature (20, 30, 40, 60 °C), of the molar amount of solketal with respect to dodecanoic acid (1.2, 1.5, 2.0 molar equivalents) and of time was carried out. This investigation of the model reaction between solketal and dodecanoic acid allowed to determine the best operative conditions. We initially established that good yields in reasonable times could be achieved with 5% PTSA, without added solvents at 60 °C. Next, we investigated the reaction progress as a function of time and of the molar amount of solketal respect to the FFA (Figure 1). Based on the trends of the figure, we chose for all further reactions a molar ratio solketal:FFA = 1.5 as this allowed to reach > 90% yield of the desired product in 4 h. From Figure 1 it is evident that a molar ratio = 1.2 is insufficient, while 2.0 causes only a 2% increase in yield over the same time, that we deemed inconsequential. In summary for the remainder of the study we used the following reaction conditions: T = 60 °C, time = 4 h, 2.0 g FFA, 1.5 molar equivalents of solketal or GlyF and 5% *w*/*w* of PTSA with respect to the FFA.

### 2.1 Synthesis of the Fatty Acid Solketal Esters (FASEs)

Four different free fatty acids (RCH_2_CO_2_H, R = C_6_H_13_, C8:0; C_10_H_21_, C12:0; C_14_H_29_, C16:0; C_16_H_33_, C18:0) were reacted with 1.5 molar equivalents of solketal in the presence of 5 wt % of PTSA at 60 °C and without added solvents (Equation (1)).

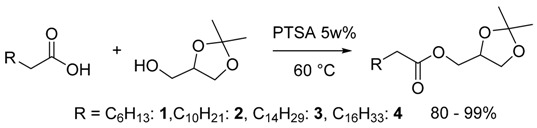
(1)

After 4 h, complete conversion of the FFA was observed by GC analysis. The results are summarized in Table 1: the reactions were 100% selective towards the formation of the fatty acid solketal esters (FASEs) **1**–**4** that were purified by FCC and obtained with high yields (80%–99%) and high purity. The products were characterized by IR, GC/MS and ^1^H-, ^13^C-NMR spectroscopy and by thermal analysis (DSC).

It is significant that the reaction could be conducted in the absence of added solvents, *i.e.* using just 1.5 molar equivalents of solketal itself as the reaction medium. This feature is desirable in view of developing more efficient and less waste-generating processes. No other products such as mono-glycerides deriving from ring opening were detected by GC-MS analysis of the crude reaction mixture and in the NMR spectrum of the isolated product, thus ensuring that no acetal ring-opening occurred, notwithstanding the acidic conditions determined by the PTSA catalyst as well as by the FFA itself.

**Table 1:** Reactions between solketal and FFAs ^a^.

FFA	mol × 10^−3^	Solketal ^b^ (mol × 10^−3^)	PTSA ^c^ (g)	Product	Isolated Yield (%)	Purity (% GC)	Melting Point (°C)
C8:0	13.80	20.7	0.1	**1**	92	> 99	liquid
C12:0	9.98	15.0	0.1	**2**	80	97	40–48
C16:0	7.79	11.7	0.1	**3**	> 99	92	55
C18:0	7.03	10.5	0.1	**4**	87	>99	54

^a^ Conditions: T = 60 °C, time = 4 h, 2.0 g FFA; ^b^ solketal 1.5 mol eq with respect to FFA; ^c^ *p*-toluensulfonic acid: 5% *w*/*w* of FFA.

DSC scans of compounds **2**–**4**—*i.e.*, the solid solketal esters of the fatty acids—were acquired in order to determine their melting points (see the supplementary materials). Two consecutive cooling and heating cycles were completed for each compound in order to exclude decomposition and to ensure reproducibility. Interestingly, the C_16_- and C_18_-solketal analogs **3** and **4** each showed two thermal transitions upon heating. Namely, (2,2-dimethyl-1,3-dioxolan-4-yl)methyl palmitate (**3**) showed a first endothermic peak at 30 °C followed by melting at 55 °C (Figure S12); while (2,2-dimethyl-1,3-dioxolan-4-yl)methyl stearate (**4**) showed a first endothermic peak at 39 °C followed by melting at 54 °C (Figure S16). This behavior indicated that compounds **3** and **4** were thermotropic and likely possessed a liquid-crystal-like mesophase in the interval between the two endothermic transitions. Given the amphiphilic structure of these molecules our hypothesis of liquid-crystalline behavior is supported by analogous thermotropic liquid crystalline behavior of a similar dodecyloxy-substituted polyol [36]. Specifically, 1-*O*-dodecylpropanetriol was shown to have a smectic phase in the temperature interval 32–48 °C, very similar to the intervals observed in our case for **3** and **4**. Additional support for the liquid crystal behavior came also by the evidence that analogous glycol-glycerol-lipid compounds also showed comparable mesogenic properties [37]. Unlike **3** and **4**, upon heating, the C_12_ fatty acid derivative (2,2-dimethyl-1,3-dioxolan-4-yl)methyl dodecanoate (**2**) showed only one broad transition around its melting point between 40 and 48 °C (Figure S8). The apparent reason why compound **2** did not show two endothermic transitions as did **3** and **4** may possibly be due to its narrow liquid crystalline interval and to the limited resolution of the DSC trace of Figure S8.

### 2.2 Synthesis of the Fatty Acid Glycerol Formal Esters (FAGEs)

The same four different free fatty acids as above were reacted with 1.5 molar equivalents of GlyF under the same operative conditions (Equation (2)).

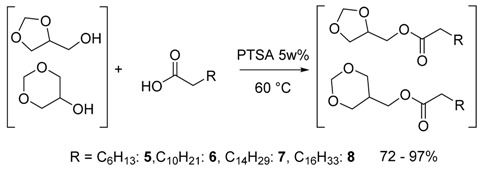
(2)

The results are summarized in Table 2: after 4 h, complete conversion of the FFA was observed by GC analysis and the reactions were 100% selective towards the formation of the fatty acid GlyF esters (FAGEs) **5**–**8** that were purified by FCC and obtained with high yields (72%–97%) and high purity. Not even traces of the ring-opening products derived from deacetalization were observed. The products were characterized by IR, GC/MS and ^1^H-, ^13^C-NMR spectroscopy and by thermal analysis (DSC).

The DSC traces of compounds **6**–**8**, *i.e.*, the solid GlyF esters of the fatty acids, all showed one single endothermal peak in correspondence with their liquid to solid transition. Unlike the corresponding solketal derivatives **3** and **4** that showed mesogenic behavior, the single liquid-solid transition of the GlyF derivatives **6**–**8** was ascribed to impaired packing of these compounds due to their existence as a mixture of the five- and six-membered acetals rings.

**Table 2:** Reaction between glycerol formal and the FFAs ^a^.

FFA	mol × 10^−3^	GlyF ^b^ (mol × 10^−3^)	PTSA^c^ (g)	Product	Isolated Yield (%)	Purity (% GC)	Melting Point (°C)
C8:0	13.80	20.7	0.1	5	72	86	liquid
C12:0	9.98	15.0	0.1	6	91	76	35–38
C16:0	7.79	11.7	0.1	7	97	97	51–54
C18:0	7.03	10.5	0.1	8	97	> 99	66–70

^a^ Conditions: T = 60 °C, time = 4 h, 2.0 g FFA; ^b^ GlyF 1.5 mol eq with respect to FFA; ^c^ *p*-toluensulfonic. acid: 5% *w*/*w* of FFA.

## 3. Experimental Section

### 3.1 Materials and Methods

All reagents and analytical grade solvents are commercially available and were used as received. Solketal ((±)-2,2-dimethyl-1,3-dioxolane-4-methanol, (±)-2,2-dimethyl-4-hydroxymethyl-1,3-dioxolane, 1,2-isopropylidene-*rac*-glycerol); glycerol formal (4-hydroxymethyl-1,3-dioxolane); *p*-toluenesulfonic acid monohydrate (ACS reagent, ≥98.5); caprylic acid (octanoic acid, C8:0); lauric acid (dodecanoic acid C12:0); palmitic acid (hexadecanoic acid C16:0); stearic acid (octadecanoic acid C18:0) were purchased from Sigma-Aldrich (Italy). Here we describe the general synthetic procedure used for the synthesis of 2,2-dimethyl-1,3-dioxolan-4-yl)methyl dodecanoate as an example of fatty acid solketal esters (FASE) and of 1,3-dioxolan-4-yl)methyl dodecanoate as an example of fatty acid glycerol formal esters (FAGE). All experimental and characterization details of the other products can be found in the Supplementary Materials.

### 3.2 Characterization 

All the ^1^H- and ^13^C-NMR spectra were collected at 25 °C on a Unity spectrometer operating at the frequency of 400 MHz for ^1^H and at 100 MHz for ^13^C (Varian, Palo Alto, CA, USA). Chemical shifts (δ) are reported in ppm relative to residual solvent signals for ^1^H- and ^13^C-NMR (^1^H-NMR: 7.26 ppm for CDCl_3_; ^13^C-NMR: 77.0 ppm for CDCl_3_). MS spectra were acquired with an Agilent GC 6890 gas chromatograph coupled with a MS 5975 mass spectrometer (Agilent, Cernusco sul Naviglio, Italy). IR spectra were measured using a Spectrum One FT-IR Spectrometer from Perkin Elmer (Milano, Italy); FR-DTGS Detector 7800–350 cm^−1^ with beamsplitter. Calorimetric measurements were performed on a DSC 92 apparatus (Setaram Abbiategrasso, Italy): approximately 100 mg of product was placed in an aluminium crucible. An empty crucible was placed in the reference cell. The temperature scanning rate was 2 °C/min.

### 3.3 General Procedure for Synthesis of Fatty Acid Solketal Esters (FASEs)

The free fatty acid (2.0 g), solketal (1.5 molar equivalent respect to the free fatty acid) and *p*-toluenesulfonic acid (5% *w*/*w* respect of the weight of the free fatty acid) were charged into a conventional round bottom flask with a magnetic stirrer. The mixture was heated at 60 °C and stirred vigorously for 4 h. The reaction was quenched by neutralizing the acid catalyst with a saturated solution of sodium carbonate then filtered. The liquid phase was collected in a separatory funnel and partitioned between chloroform and water (3 × 20 mL). The organic layer was dried over sodium sulfate, filtered and the solvent eliminated using a rotary evaporator. The sample was stored in a glass vial. Equation (1) and Table 1 summarize experimental conditions and results of reactions between solketal and the FFAs.

*(2,2-Dimethyl-1,3-dioxolan-4-yl)methyl octanoate* (**1**) Liquid at RT, m.p. ≈ −25 °C; ^1^H-NMR (400 MHz) 4.36–3.44 (m, 4H), 2.35 (td, *J* = 7.5, 2.2 Hz, 2H), 1.63 (tq, *J* = 10.6, 6.8, 5.2 Hz, 2H), 1.44 (t, 2H), 1.37 (t, 2H), 1.29 (dtq, *J* = 13.8, 9.0, 5.0, 4.6 Hz, 9H), 0.88 (t, *J* = 6.4 Hz, 3H); ^13^C-NMR (CDCl_3_, 100 MHz) (ppm): 173.39, 109.51, 77.38, 69.93, 65.52, 33.79, 31.31, 28.73, 28.57, 26.33, 25.05, 24.56, 22.25, 13.71; GC-MS (relative intensity, 70 eV) *m*/*z*: 83 (10), 55 (10), 69 (11), 41 (11), 116 (12), 41 (14), 116 (15), 244 (15), 43 (18), 130 (19), 117 (20), 284 (20), 131 (21), 55 (23), 129 (25), 127 (30), 57 (31), 101 (32), 43 (35), 101 (37), 243 (100), 258 ([M]^+^, <1%); IR (wavenumber cm^−1^; Transmittance %): 3458; 70, 2930; 52, 1741; 51, 1384; 61, 1164; 63, 1050; 73, 700; 80.

*(2,2-Dimethyl-1,3-dioxolan-4-yl)methyl dodecanoate* (**2**) m.p. = 40–48 °C; ^1^H-NMR (400 MHz) 4.38–3.42 (m, 4H), 2.34 (td, *J* = 7.6, 3.1 Hz, 2H), 1.63 (td, *J* = 7.3, 4.2 Hz, 3H), 1.44 (d, *J* = 4.8 Hz, 2H), 1.38 (d, *J* = 2.9 Hz, 2H), 1.26 (s, 18H), 0.88 (t, *J* = 6.6 Hz, 3H); ^13^C-NMR (100 MHz): 173.36, 109.53, 76.10, 69.98, 64.88, 33.85, 33.28, 31.61, 29.30, 29.15, 29.03, 28.95, 28.83, 26.40, 25.11, 24.61, 22.39, 13.82; GC-MS (relative intensity, 70 eV) *m*/*z*: 83 (3), 85 (3), 185 (3), 213 (3), 41 (5), 71 (5), 171 (5), 55 (6), 183 (7), 57 (8), 129 (9), 116 (12), 43 (15), 300 (18), 101 (21), 299 (100), 314 ([M]^+^, <1%); IR (wavenumber cm^−1^; Transmittance %) 3292; 20, 2920; 0, 1739; 1, 1469; 7, 1418; 20, 1380; 14, 1208; 11, 1183; 10, 1104; 15, 1048; 12, 992; 31, 943; 37, 849; 41, 757; 13, 720; 25.

*(2,2-Dimethyl-1,3-dioxolan-4-yl)methyl palmitate* (**3**) m.p. = 55 °C; ^1^H-NMR (400 MHz) 4.11 (dt, *J* = 11.5, 6.1 Hz, 2H), 2.36 (t, *J* = 7.5 Hz, 2H), 1.64 (s, 3H), 1.45 (t, 3H), 1.38 (t, 3H), 1.28 (d, *J* = 6.8 Hz, 34H), 0.89 (t, *J* = 6.2 Hz, 3H); ^13^C-NMR (100 MHz): 173.63, 109.52, 73.37, 66.07, 64.87, 33.84, 31.63, 29.38, 29.15, 29.06, 29.06, 29.06, 29.06, 28.95 28.95, 28.83, 26.40, 26.22, 25.10, 24.62, 22.41, 13.83; GC-MS (relative intensity, 70 eV) *m*/*z*: 171 (7), 55 (8), 57 (10), 116 (12), 129 (12), 43 (17), 101 (21), 356 (24), 355 (100), 370 ([M]^+^, <1%); IR (wavenumber cm^−1^; Transmittance %) 3234; 30, 2917; 6, 1730; 9, 1469; 13, 1382; 14, 1220; 13, 1048; 15, 849; 27, 757; 12, 667; 35, 516; 48.

*(2,2-Dimethyl-1,3-dioxolan-4-yl)methyl stearate* (**4**) m.p. = 54 °C;^1^H-NMR (400 MHz) δ 4.38–3.53 (m, 5H), 2.42–2.29 (m, 2H), 1.45 (dt, *J* = 2.5, 0.7 Hz, 2H), 1.39 (h, *J* = 0.7 Hz, 2H), 1.27 (s, 32H), 0.97–0.82 (m, 3H); ^13^C-NMR (100 MHz) 173.36, 109.53, 77.36, 76.88, 76.72, 76.23, 76.09, 73.39, 69.99, 66.07, 64.23, 63.04, 33.86, 31.64, 29.41, 29.17, 29.07, 28.97, 28.84, 26.40, 25.12, 24.63, 22.41, 13.83; GC-MS (relative intensity, 70 eV) *m*/*z*: 69 (3), 83 (3), 85 (3), 267 (3), 297 (4), 341 (4), 385 (4), 41 (5), 71 (5), 185 (5), 55 (6), 171 (7), 340 (7), 57 (8), 116 (9), 129 (11), 43 (14), 101 (17), 384 (25), 383 (100), 398 ([M]^+^, <1%); IR (wavenumber cm^−1^; Transmittance %) 3234; 26, 2916; 0, 2890; 0, 1730; 2, 1472; 1, 1418; 23, 1381; 10, 1292; 25, 1216, 3; 1180; 5, 1048; 6, 991; 36, 944; 42, 848; 33, 758; 3, 719; 17, 667; 52, 515; 60.

### 3.4 General Procedure for Synthesis of Fatty Acid Glycerol Formal esters (FAGE)

The FFA (2.0 g), glycerol formal (GlyF: 1.5 molar equivalent respect to the FFA) and *p*-toluene-sulfonic acid (5% *w*/*w* respect of the weight of the FFA) were charged in a conventional round bottom flask with a magnetic stirrer. The mixture was heated at 60 °C and stirred vigorously for 4 h. The reaction was quenched by neutralizing the acid catalyst with saturated solution of sodium carbonate then filtered. The liquid phase was collected in a separatory funnel and partitioned between chloroform and water (3 × 20 mL). The organic layer was dried over sodium sulfate, filtered and the solvent eliminated using a rotary evaporator. The sample was stored in a glass vial Table 2 summarizes the experimental conditions and the results of reactions between GlyF and the FFAs.

*(1,3-Dioxolan-4-yl)methyl octanoate* (**5**) Liquid at RT; ^1^H-NMR (400 MHz, CDCl_3_) δ 4.35–3.41 (m, 4H), 2.35 (ddd, *J* = 9.8, 6.6, 2.2 Hz, 2H), 1.63 (depth, *J* = 11.1, 3.5 Hz, 2H), 1.44 (s, 2H), 1.29 (d, *J* = 6.0 Hz, 9H), 0.92–0.85 (m, 3H); ^13^C-NMR (100 MHz) δ 178.88, 95.12, 77.38, 68.26, 63.03, 33.95, 33.84, 31.32, 28.75, 28.72, 28.58, 24.57, 24.39, 22.27, 13.73; GC-MS (relative intensity, 70 eV) *m*/*z*: 69 (5), 83 (5), 116 (5), 84 (6), 109 (7), 42 (8), 58 (8), 73 (8), 126 (8), 128 (8), 98 (9), 103 (10), 45 (11), 87 (11), 145 (12), 146 (15), 41 (21), 43 (21), 55 (26), 86 (68), 57 (77), 127 (100), 230 ([M]^+^, <1%); IR (wavenumber cm^−1^; Transmittance %) 3558; 77, 2930; 32, 2858; 44, 1739; 32, 1492; 68, 1384; 61, 1166; 44, 1045; 55, 942; 64,757; 56.

*(1,3-Dioxolan-4-yl)methyl dodecanoate* (**6**) m.p. = 35–38 °C; ^1^H-NMR (400 MHz) δ 5.14–3.82 (m, 6H), 3.71 (dq, *J* = 14.0, 5.0, 4.1 Hz, 2H), 2.37 (t, *J* = 7.6 Hz, 3H), 1.65 (t, *J* = 7.3 Hz, 3H), 1.42–1.22 (m, 9H), 0.95–0.82 (m, 3H); ^13^C-NMR (100 MHz, ) δ 178.71, 93.37, 77.35, 68.29, 63.59, 33.97, 29.30, 29.03, 28.94, 28.82, 28.77, 26.39, 24.60, 24.40, 22.38, 13.81; GC-MS (relative intensity, 70 eV) *m*/*z*: 56 (10), 184 (10), 58 (12), 112 (12), 97 (13), 95 (14), 201 (14), 69 (15), 83 (17), 85 (17), 45 (19), 71 (19), 84 (21), 87 (23), 73 (24), 146 (24), 103 (25), 41 (33), 98 (37), 55 (39), 43 (40), 57 (56), 183 (69), 86 (100), 286 ([M]^+^, <1%); IR (wavenumber cm^−1^; Transmittance %) 3451; 62, 2926; 25, 2855; 37, 1740; 4 4, 1467; 66, 1384; 61, 1156; 57, 1117; 60, 956; 74, 556; 76.

*(1,3-Dioxolan-4-yl)methyl palmitate* (**7**) m.p. = 51–54 °C; ^1^H-NMR (400 MHz) δ 5.06–3.52 (m, 6H), 2.35 (t, *J* = 7.5 Hz, 2H), 1.64 (t, *J* = 7.3 Hz, 3H), 1.26 (s, 27H), 0.89 (t, *J* = 6.3 Hz, 3H); ^13^C-NMR (100 MHz) δ 178.71, 95.15, 77.36, 68.29, 66.43, 65.17, 33.97, 33.82, 33.59, 31.63, 29.39, 29.36, 29.30, 29.06, 28.95, 28.83, 28.77, 24.60, 24.40, 22.40, 13.82; GC-MS (relative intensity, 70 eV) *m*/*z*: 67 (10), 341 (10), 56 (11), 81 (11), 129 (11), 257 (11), 95 (13), 111 (13), 58 (18), 85 (18), 45 (19), 146 (19), 112 (21), 69 (22), 83 (22), 71 (23), 97 (24), 84 (32), 41 (33), 87 (37), 55 (38), 103 (38), 73 (43), 43 (44), 239 (52), 57 (63), 98 (64), 86 (100), 342 ([M]^+^, <1%); IR (wavenumber cm^−1^; Transmittance %) 2916; 1, 2850; 2, 1738; 7, 1704; 11, 1463; 19, 1383; 43, 1297; 29, 1175; 17, 1097; 25, 1015; 27, 943; 23, 758; 11, 720; 40, 667; 63.

*(1,3-Dioxolan-4-yl)methyl stearate* (**8**) m.p. = 66–70 °C; ^1^H-NMR (400 MHz) δ 5.16–3.53 (m, 4H), 2.37 (s, 1H), 1.64 (s, 1H), 1.28 (d, *J* = 7.1 Hz, 25H), 0.97–0.82 (m, 3H); ^13^C-NMR (100 MHz) δ 175.83, 93.76, 74.68, 71.63, 66.89, 65.04, 63.77, 62.18, 32.58, 32.42, 32.01, 28.01, 27.97, 27.91, 27.76, 27.56, 27.44, 27.39, 23.21, 23.05, 13.57, 12.42; GC-MS (relative intensity, 70 eV) *m*/*z*: 129 (8), 268 (8), 369 (8), 96 (9), 99 (9), 109 (9), 67 (10), 81 (11), 159 (11), 285 (11), 56 (12), 111 (12), 95 (13), 58 (17), 45 (19), 112 (19), 97 (20), 85 (22), 71 (23), 69 (24), 83 (24), 146 (25), 103 (27), 84 (28), 41 (30), 73 (33), 87 (36), 267 (40), 55 (41), 43 (55), 98 (56), 57 (59), 86 (100), 370 ([M]^+^, <1%); IR (wavenumber cm^−1^; Transmittance %) 2977; 32, 2927; 32, 2855; 28, 1737; 66. 1472; 66, 1383; 49, 1350; 65, 1122; 21, 953; 80, 830; 79, 680; 77.

## 4. Conclusions

We have presented the acid-catalyzed synthesis and characterization of the fatty acid esters of solketal and glycerol formal. The novelty lies in the fact that acid catalysis does not promote undesired acetal hydrolysis and that the procedure can be conducted in the absence of added solvents. In fact, it is likely that the absence of added solvents, normally containing traces of water, is one of the reasons for the observed lack of hydrolysis of the acetals. High yields of isolated products can be obtained and all synthesized compounds are stable. In addition, the derivatives of solketal **3** and **4** possess mesogenic behavior and show a liquid crystalline phase. These new products contain different carbon-oxygen moieties that can in principle provide interesting properties as fuel additives such as better ignition and/or lubricity.

## Supplementary Materials

Supplementary materials can be accessed at: http://www.mdpi.com/1420-3049/21/2/170/s1.
