# Supplementary material for: Synthesis of the Fatty Esters of Solketal and Glycerol-Formal: Biobased Specialty Chemicals
Source: Molecules. 2016 Jan 30;21(2):170. doi: 10.3390/molecules21020170 (PMC6273257; doi:10.3390/molecules21020170)
Supplement: Supplementary file 1 [file molecules-21-00170-s001.pdf]

Supplementary Materials: Synthesis of the Fatty Esters of Solketal and Glycerol-Formal: Biobased Specialty Chemicals

Alvise Perosa, Andrea Moraschini, Maurizio Selva and Marco Noè

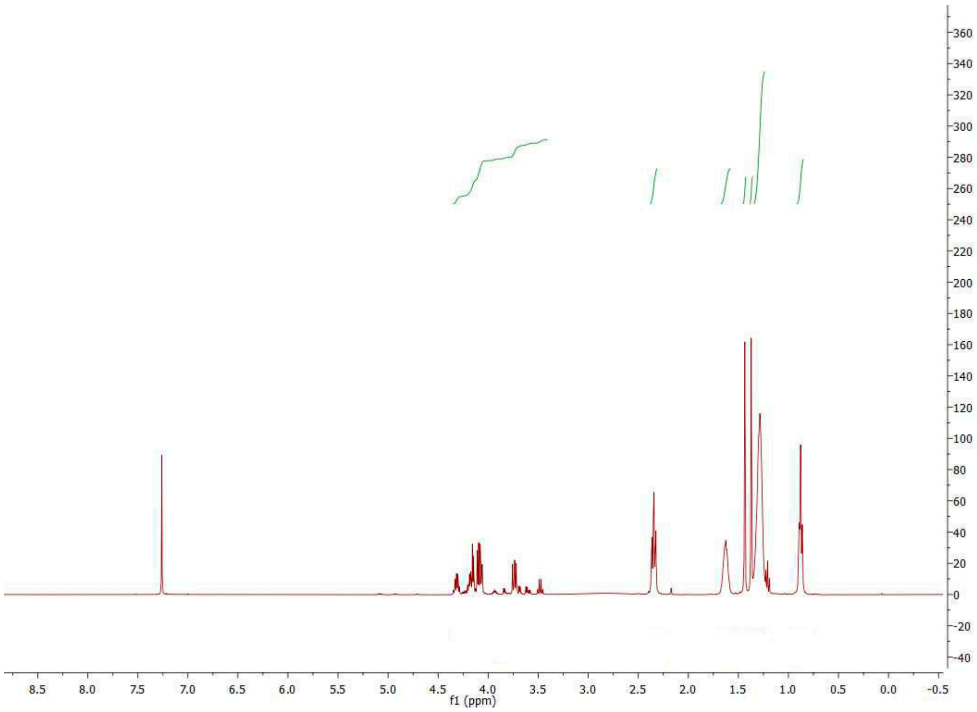

Figure S1. ^{1}H-NMR spectrum of product **1**: (2,2-dimethyl-1,3-dioxolan-4-yl)methyl octanoate **1**; ^{1}H-NMR (400 MHz) 4.36–3.44 (m, 4H), 2.35 (td, *J* = 7.5, 2.2 Hz, 2H), 1.63 (tq, *J* = 10.6, 6.8, 5.2 Hz, 2H), 1.44 (t, 2H), 1.37 (t, 2H), 1.29 (dtq, *J* = 13.8, 9.0, 5.0, 4.6 Hz, 9H), 0.88 (t, *J* = 6.4 Hz, 3H).

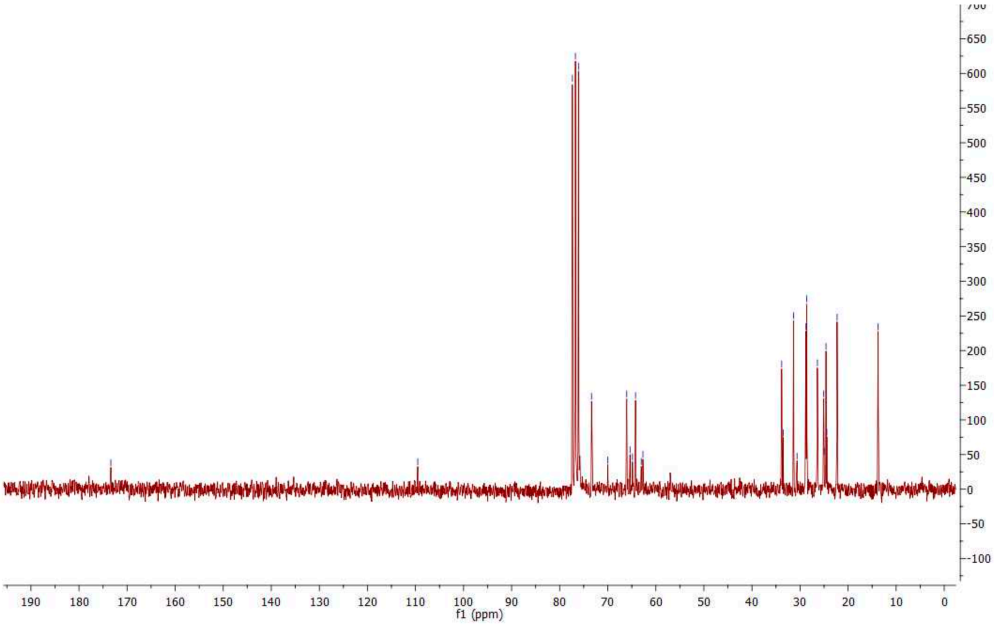

Figure S2. ^{13}C-NMR spectrum of Product **1**: (2,2-dimethyl-1,3-dioxolan-4-yl)methyl octanoate **1**: ^{13}C-NMR ($CDCl_3$, 200 MHz) (ppm): 173.39, 109.51, 77.38, 69.93, 65.52, 33.79, 31.31, 28.73, 28.57, 26.33, 25.05, 24.56, 22.25, 13.71

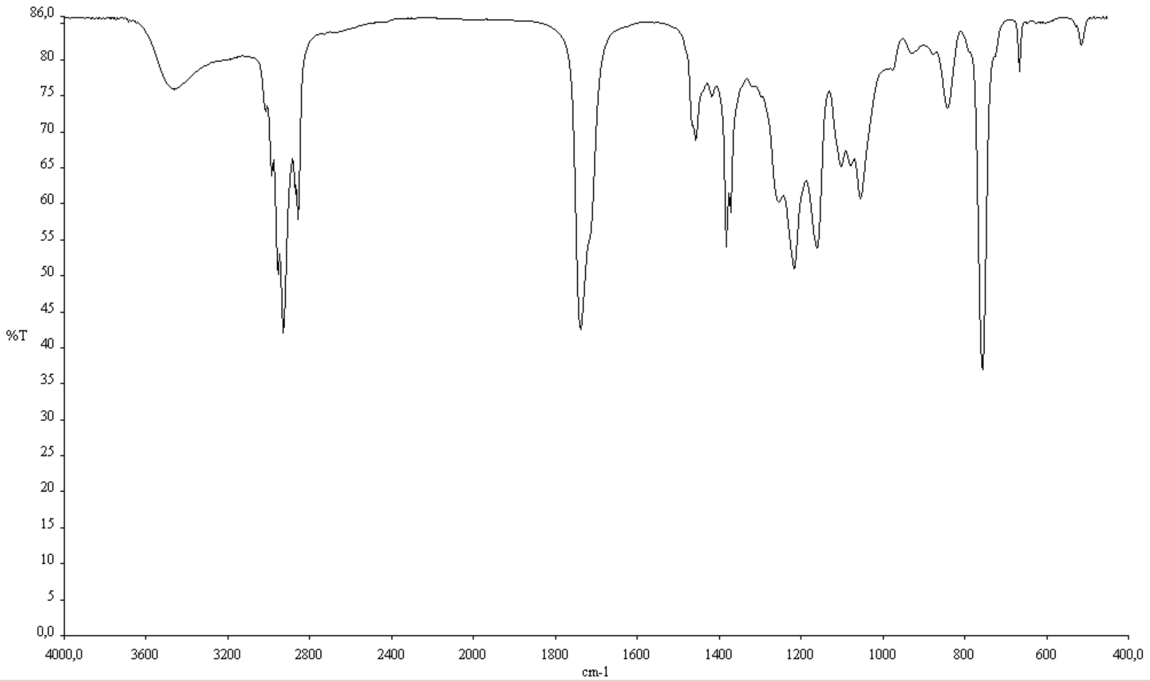

Figure S3. FT IR spectrum of product **1**: (2,2-dimethyl-1,3-dioxolan-4-yl)methyl octanoate **1**: IR (wavenumber cm^{-1}; Transmittance %): 3458;70, 2930;52, 1741;51, 1384;61, 1164;63, 1050;73, 700;80.

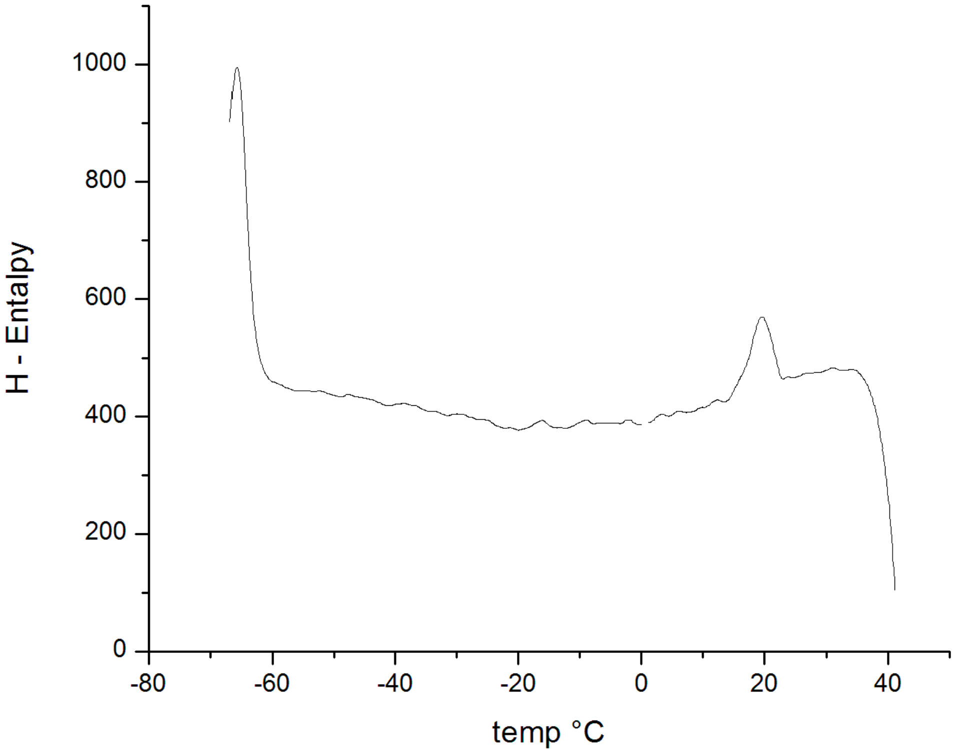

Figure S4. DSC (2,2-dimethyl-1,3-dioxolan-4-yl)methyl octanoate **1**.

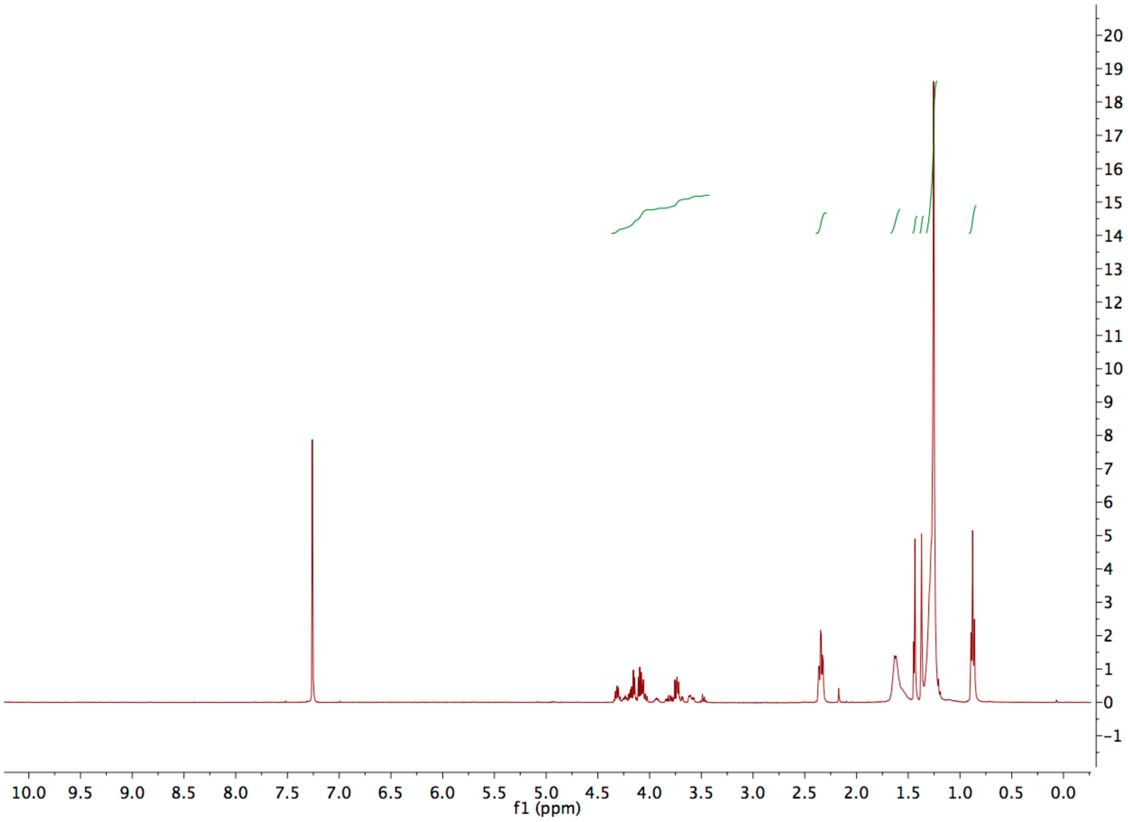

Figure S5. ^{1}H-NMR (200 MHz) spectrum of (2,2-dimethyl-1,3-dioxolan-4-yl)methyl dodecanoate **2**: ^{1}H-NMR (200 MHz) δ 5.14–3.82 (m, 6H), 3.71 (dq, *J* = 14.0, 5.0, 4.1 Hz, 2H), 2.37 (t, *J* = 7.6 Hz, 3H), 1.65 (t, *J* = 7.3 Hz, 3H), 1.42–1.22 (m, 9H), 0.95–0.82 (m, 3H).

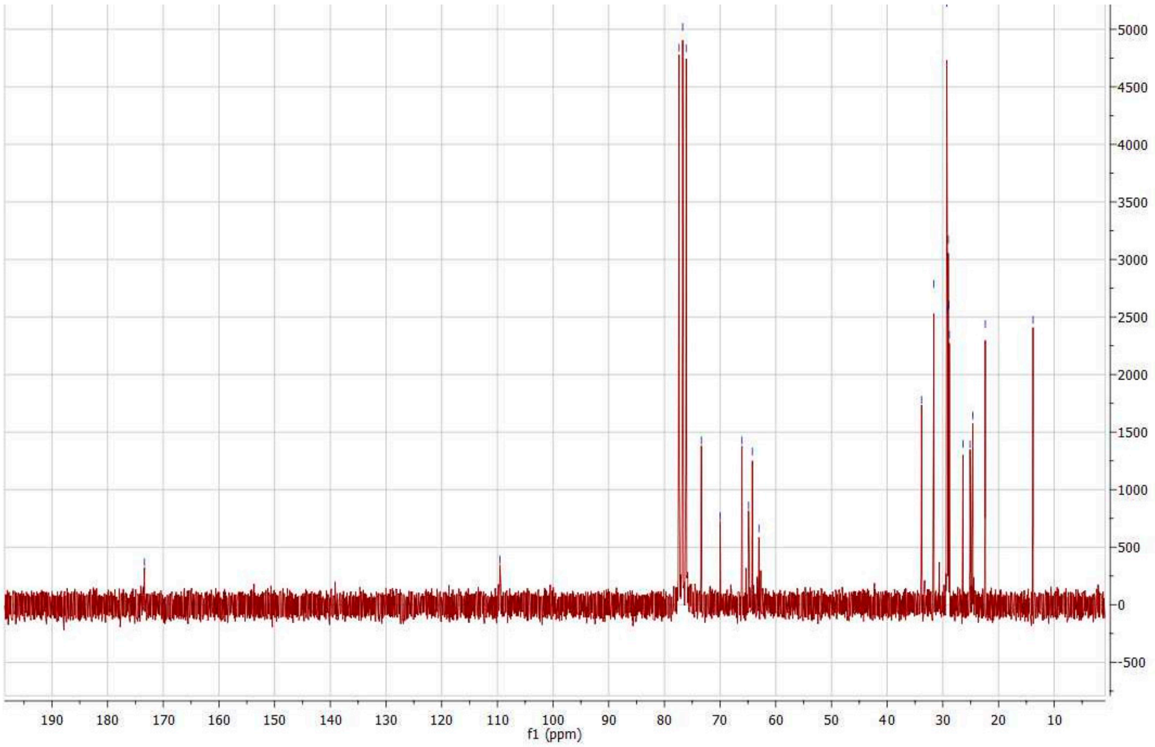

Figure S6. Spectrum of (2,2-dimethyl-1,3-dioxolan-4-yl)methyl dodecanoate **2**: ^{13}C-NMR (200 MHz): 173.36, 109.53, 76.10, 69.98, 64.88, 33.85, 33.28, 31.61, 29.30, 29.15, 29.03, 28.95, 28.83, 26.40, 25.11, 24.61, 22.39, 13.82.

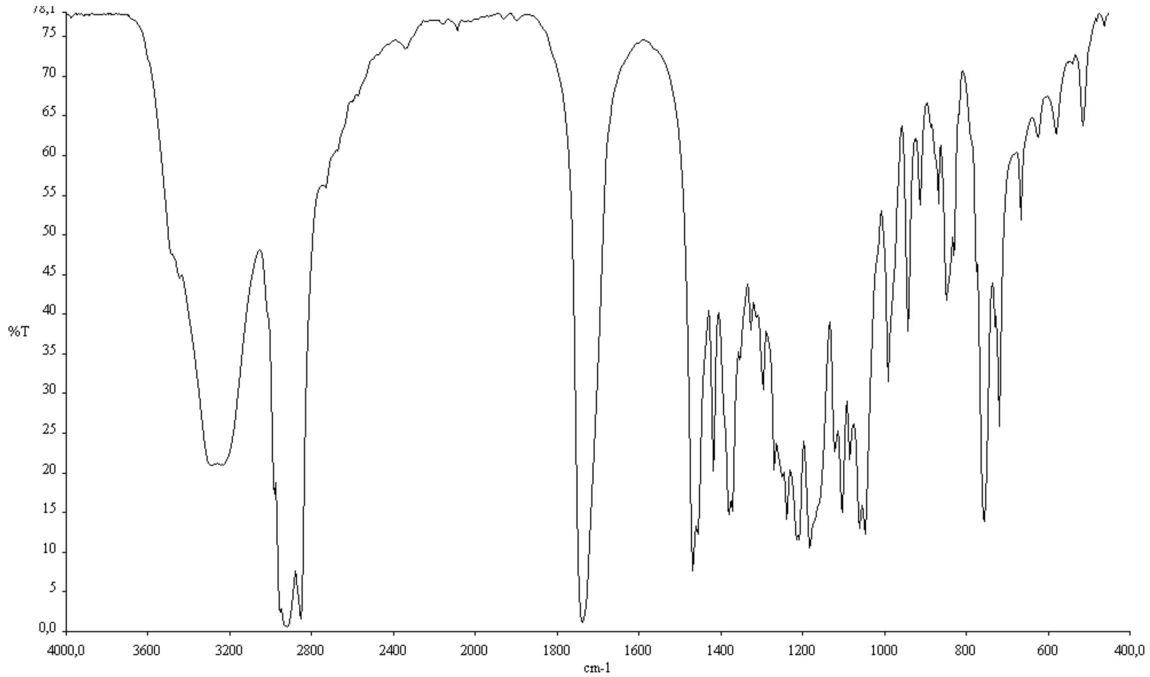

Figure S7. FT-IR spectrum of (2,2-dimethyl-1,3-dioxolan-4-yl)methyl dodecanoate **2**: IR (wavenumber cm^{-1}; Transmittance %) 3451;62, 2926;25, 2855;37, 1740;44, 1467;66, 1384;61, 1156;57, 1117;60, 956;74, 556;76.

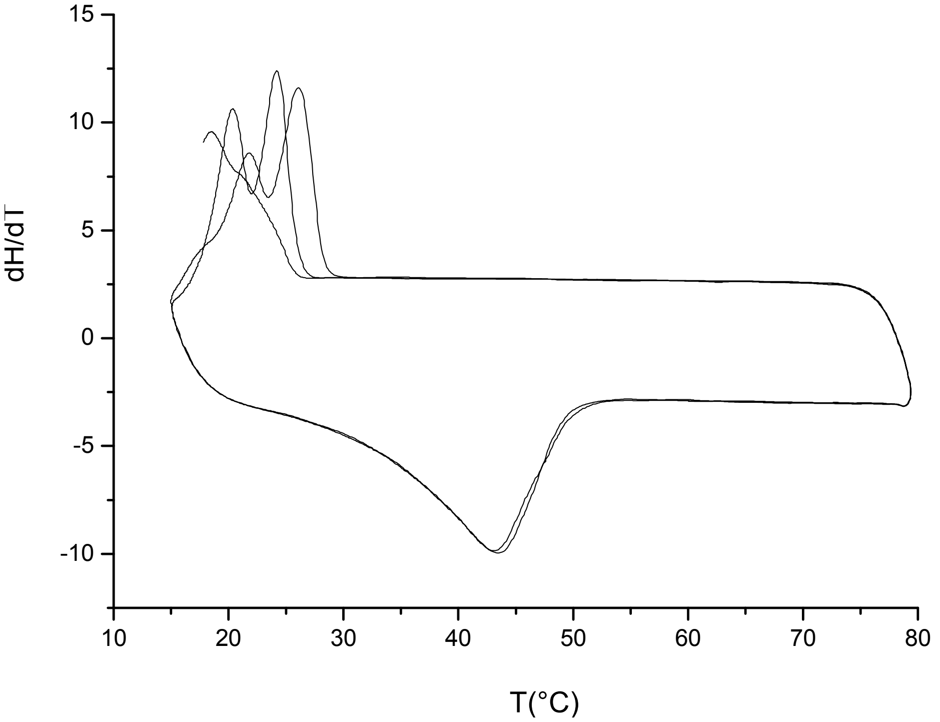

Figure S8. DSC (2,2-dimethyl-1,3-dioxolan-4-yl)methyl dodecanoate **2**.

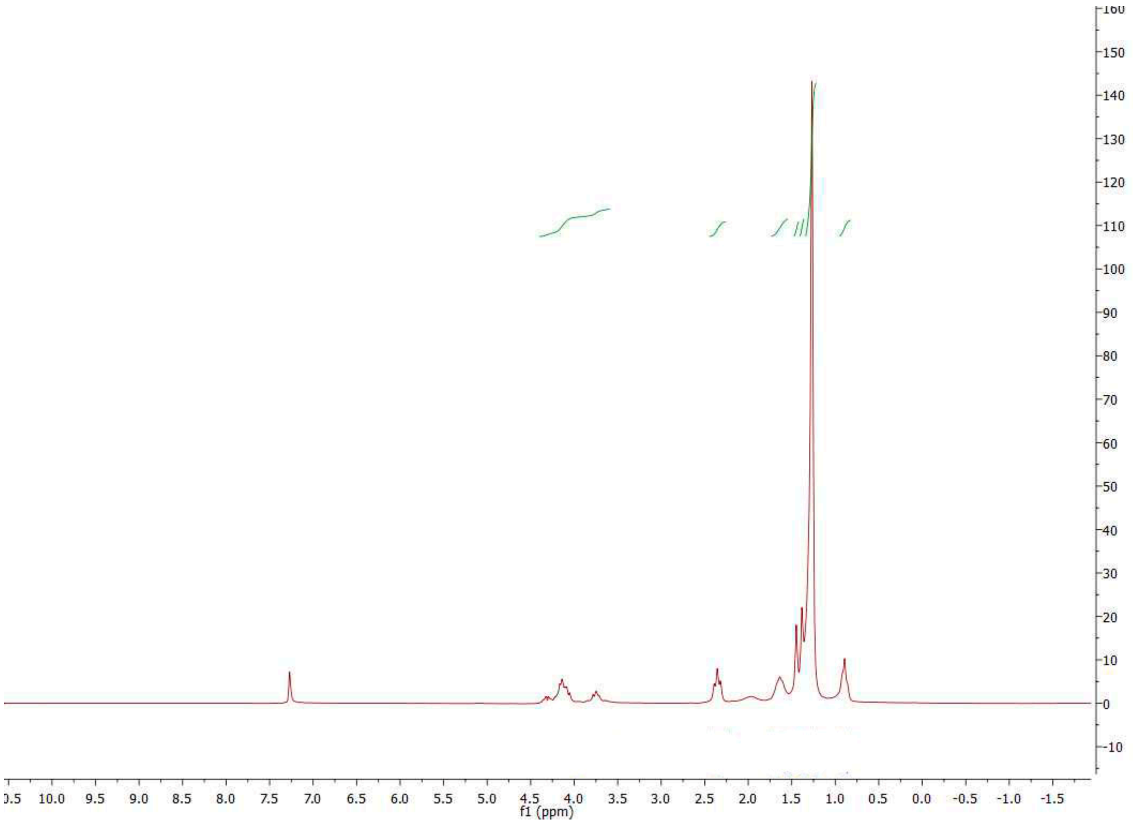

Figure S9. ^{1}H-NMR (200 MHz) spectrum of (2,2-dimethyl-1,3-dioxolan-4-yl)methyl palmitate **3**: ^{1}H-NMR (200 MHz) 4.11 (dt, *J* = 11.5, 6.1 Hz, 2H), 2.36 (t, *J* = 7.5 Hz, 2H), 1.64 (s, 3H), 1.45 (t, 3H), 1.38 (t, 3H), 1.28 (d, *J* = 6.8 Hz, 34H), 0.89 (t, *J* = 6.2 Hz.

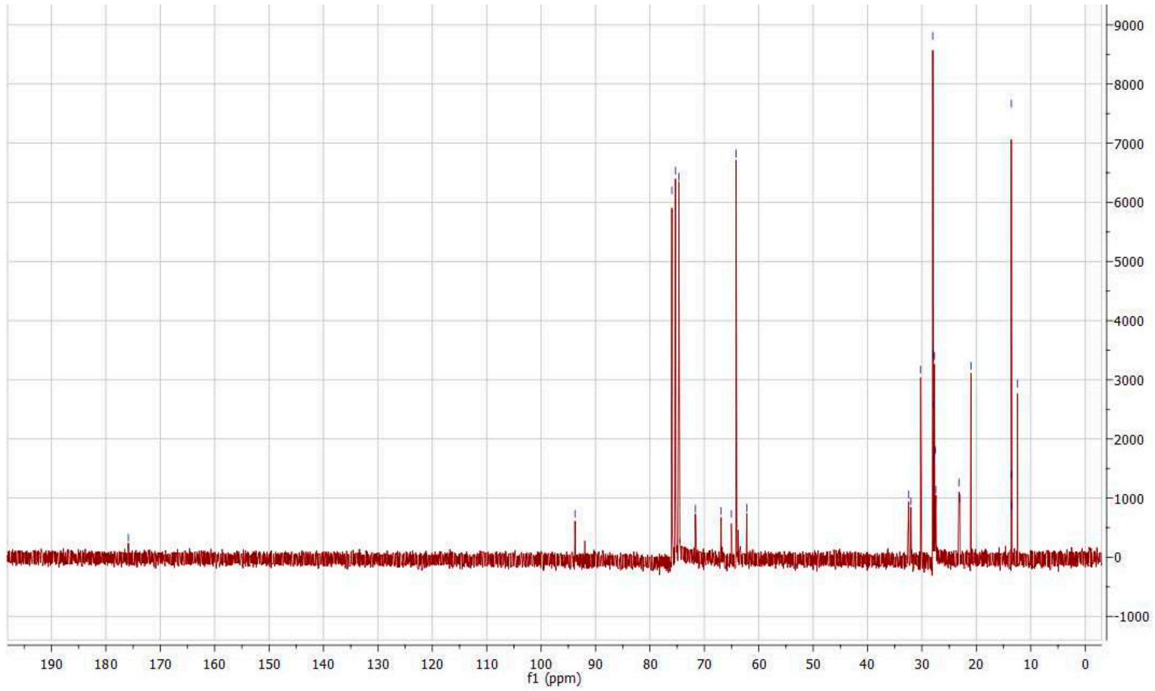

Figure S10. ^{13}C-NMR (200 MHz) spectrum of (2,2-dimethyl-1,3-dioxolan-4-yl)methyl palmitate **3**: ^{13}C-NMR (200 MHz): 173.63, 109.52, 73.37, 66.07, 64.87, 33.84, 31.63, 29.38, 29.15, 29.06, 29.06, 29.06, 29.06, 28.95 28.95, 28.83, 26.40, 26.22, 25.10, 24.62, 22.41, 13.83.

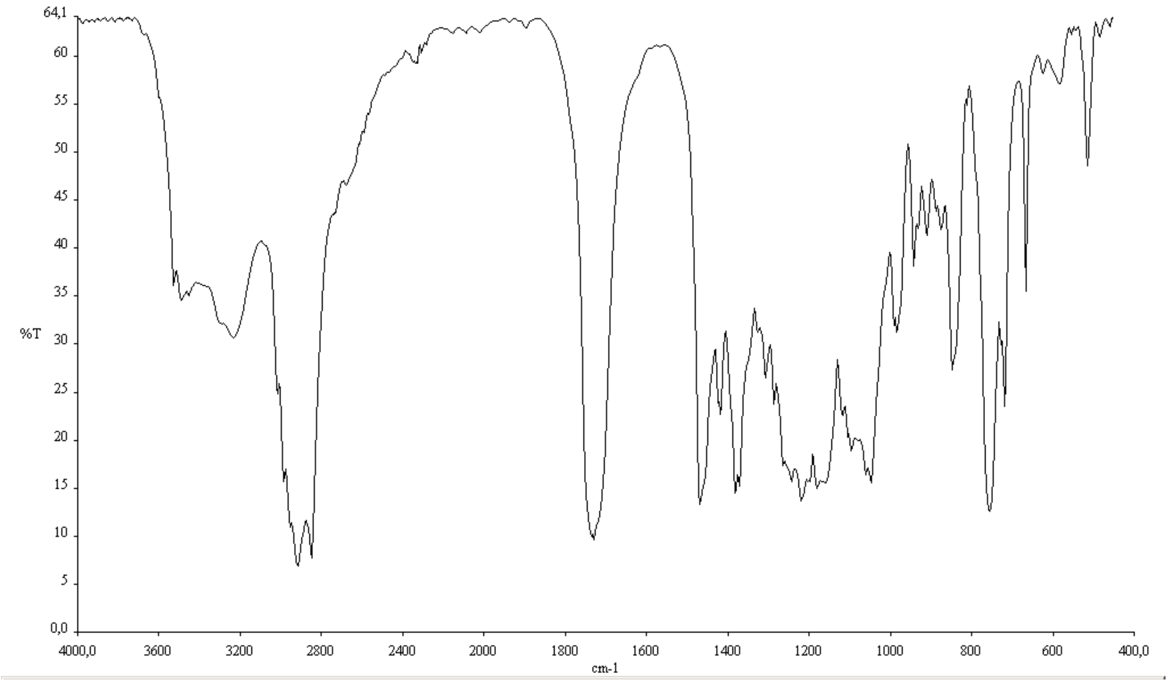

Figure S11. FT-IR spectrum of (2,2-dimethyl-1,3-dioxolan-4-yl)methyl palmitate **3**: IR (wavenumber cm^{-1}; Transmittance %) 3234;30, 2917;6, 1730;9, 1469;13, 1382;14, 1220;13, 1048;15, 849;27, 757;12, 667;35, 516;48.

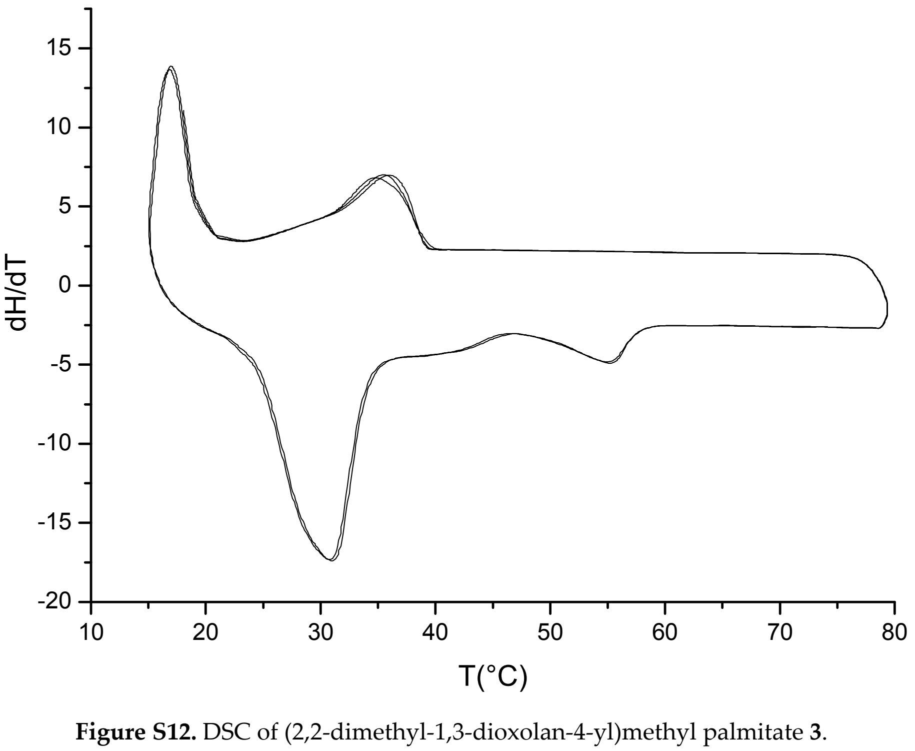

Figure S12. DSC of (2,2-dimethyl-1,3-dioxolan-4-yl)methyl palmitate **3**.

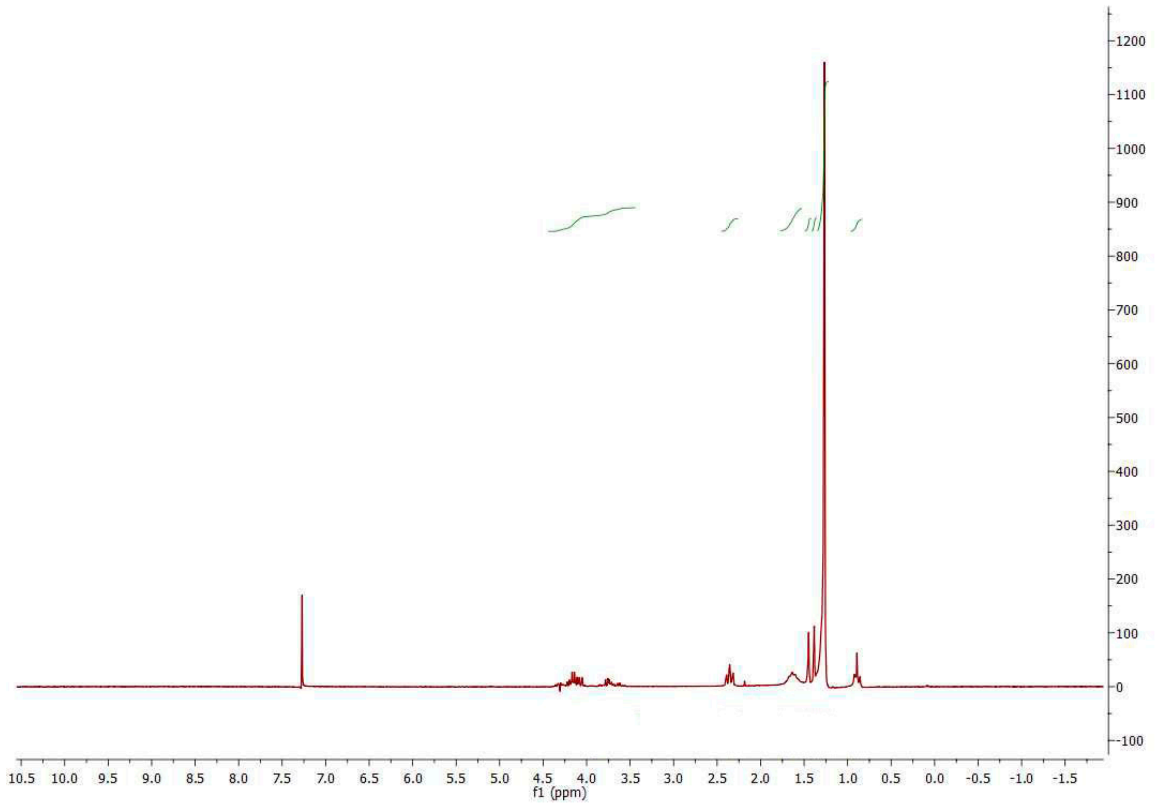

Figure S13. ^{1}H-NMR (200 MHz) spectrum of (2,2-dimethyl-1,3-dioxolan-4-yl)methyl stearate **4**: ^{1}H-NMR (200 MHz) δ 4.38–3.53 (m, 5H), 2.42–2.29 (m, 2H), 1.45 (dt, *J* = 2.5, 0.7 Hz, 2H), 1.39 (h, *J* = 0.7 Hz, 2H), 1.27 (s, 32H), 0.97–0.82 (m, 3H).

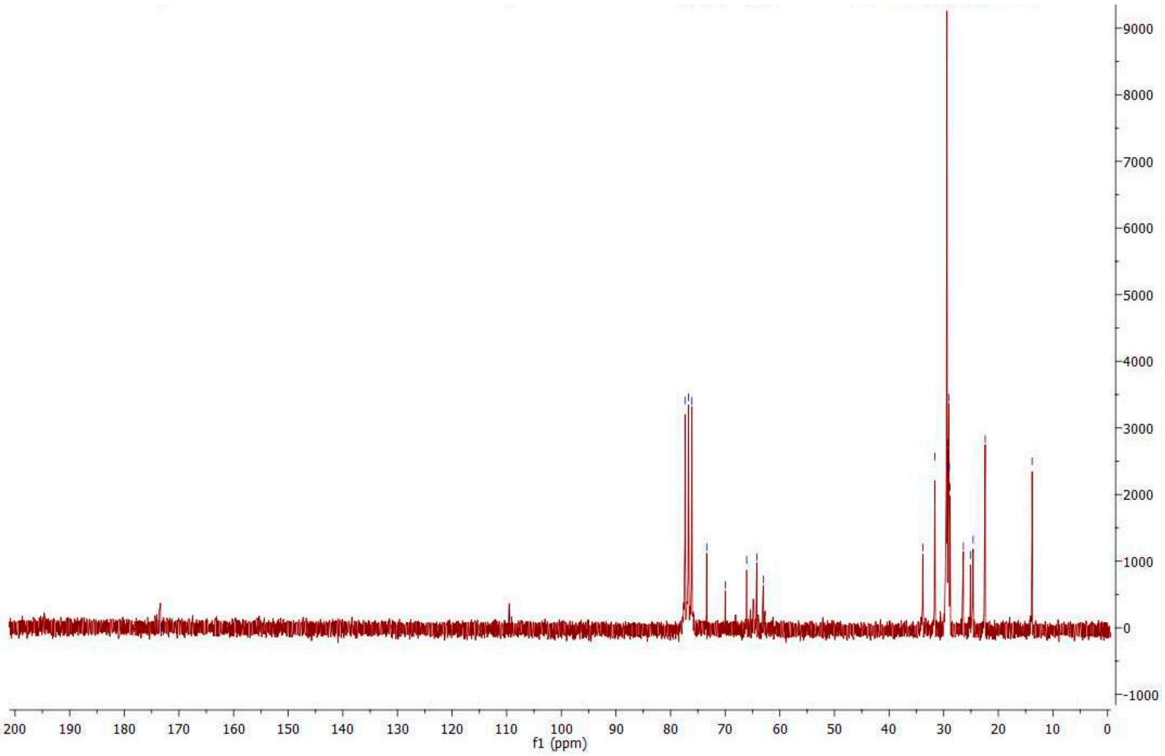

Figure S14. ^{13}C-NMR (200 MHz) spectrum of (2,2-dimethyl-1,3-dioxolan-4-yl)methyl stearate 4: ^{13}C-NMR (200 MHz) 173.36, 109.53, 77.36, 76.88, 76.72, 76.23, 76.09, 73.39, 69.99, 66.07, 64.23, 63.04, 33.86, 31.64, 29.41, 29.17, 29.07, 28.97, 28.84, 26.40, 25.12, 24.63, 22.41, 13.83.

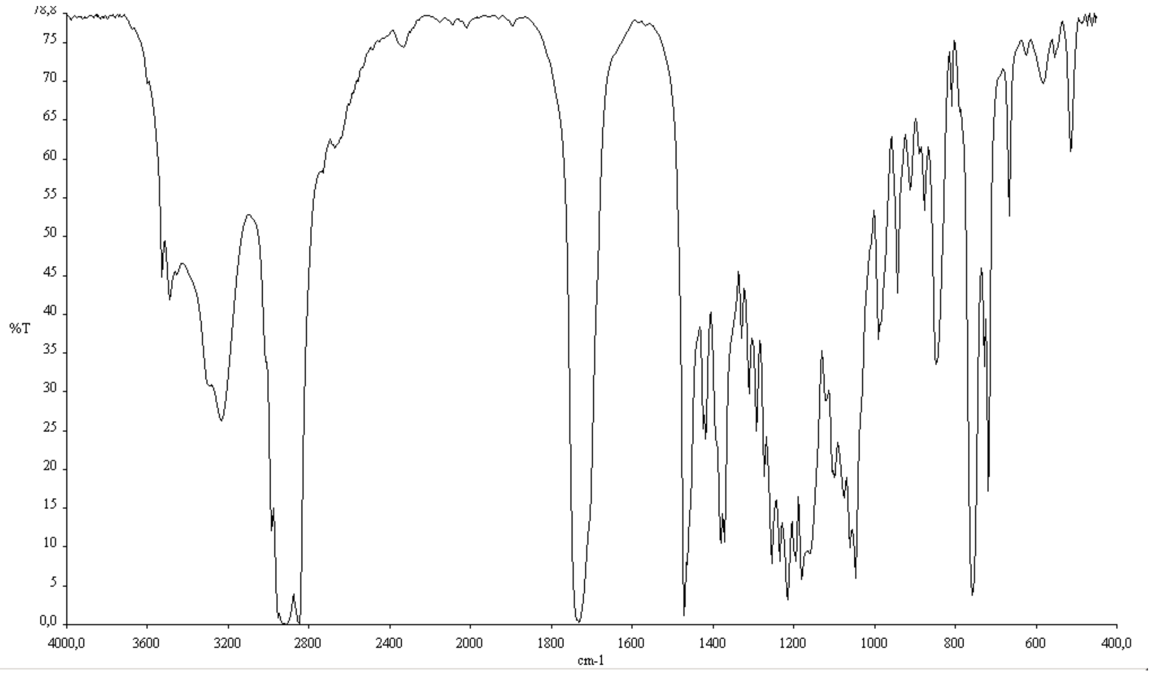

Figure S15. FT-IR spectrum of (2,2-dimethyl-1,3-dioxolan-4-yl)methyl stearate **4**: IR (wavenumber cm^{-1}; Transmittance %) 3234;26, 2916;0, 2890;0, 1730;2, 1472;1, 1418;23, 1381;10, 1292;25, 1216,3; 1180;5, 1048;6, 991;36, 944;42, 848;33, 758;3, 719;17, 667;52, 515;60.

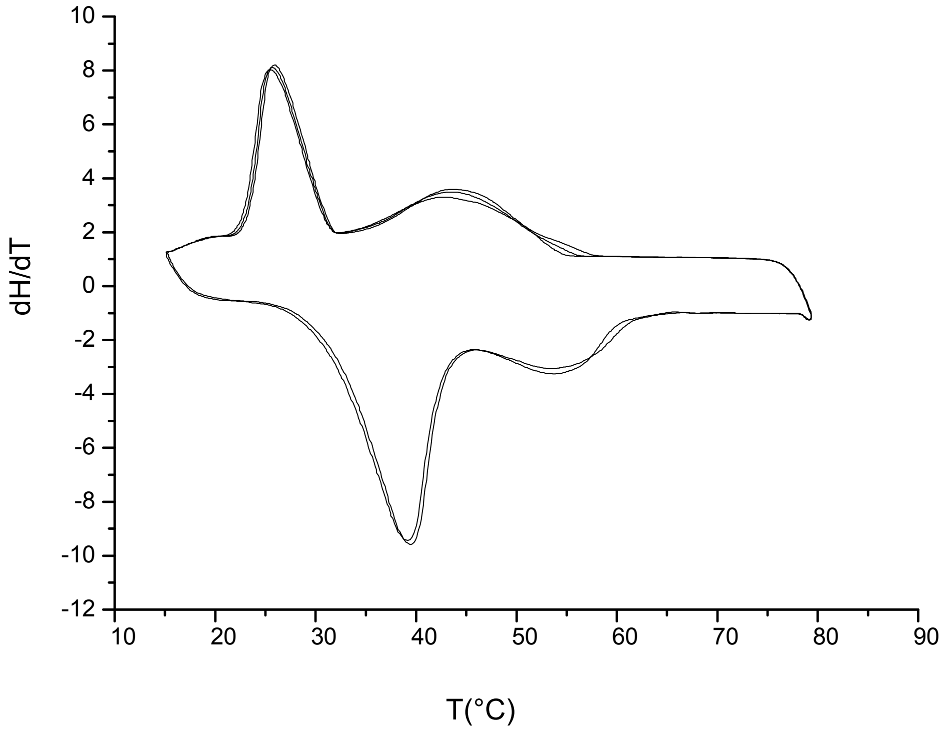

Figure S16. DSC of (2,2-dimethyl-1,3-dioxolan-4-yl)methyl stearate **4**.

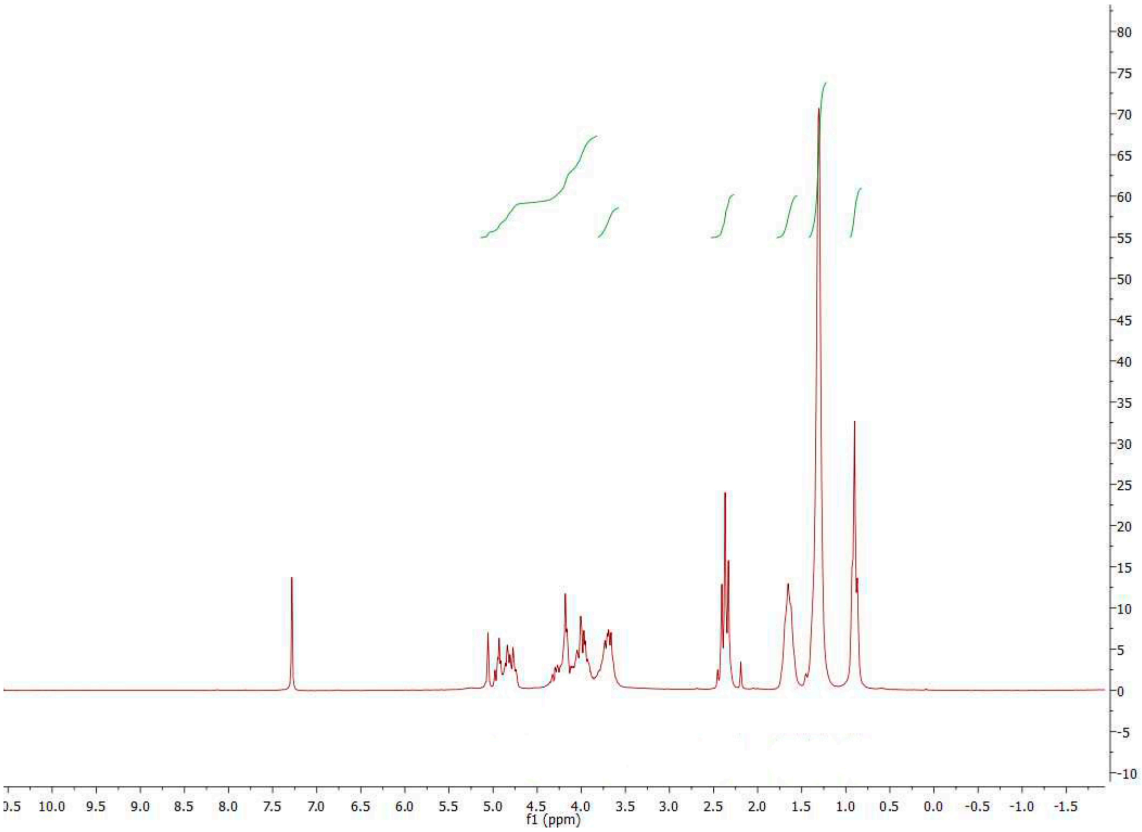

Figure S17. ^{1}H-NMR spectrum of product (1,3-dioxolan-4-yl)methyl octanoate **5**: ^{1}H-NMR (400 MHz, $CDCl_3$) δ 4.35–3.41 (m, 4H), 2.35 (ddd, *J* = 9.8, 6.6, 2.2 Hz, 2H), 1.63 (depth, *J* = 11.1, 3.5 Hz, 2H), 1.44 (s, 2H), 1.29 (d, *J* = 6.0 Hz, 9H), 0.92–0.85 (m, 3H).

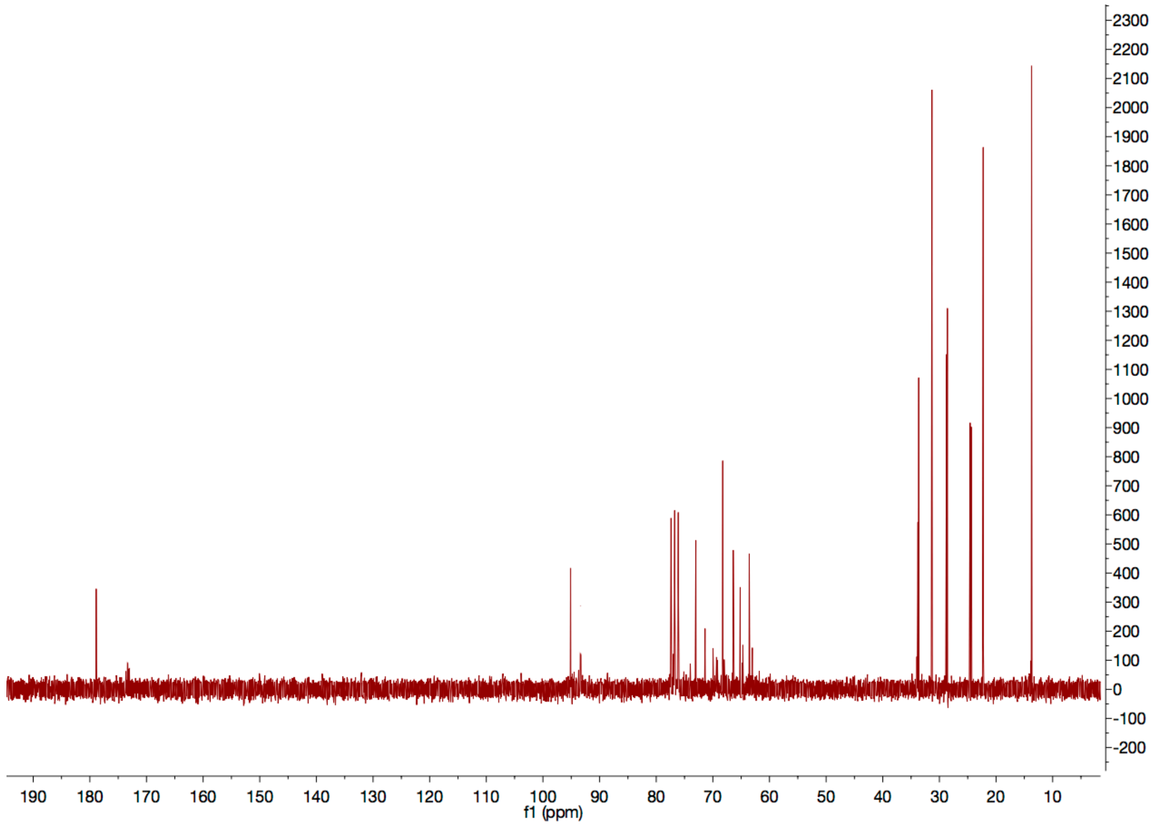

Figure S18. ^{13}C-NMR (50 MHz) of (1,3-dioxolan-4-yl)methyl octanoate **5**: ^{13}C-NMR (200 MHz) δ 178.88, 95.12, 77.38, 68.26, 63.03, 33.95, 33.84, 31.32, 28.75, 28.72, 28.58, 24.57, 24.39, 22.27, 13.73

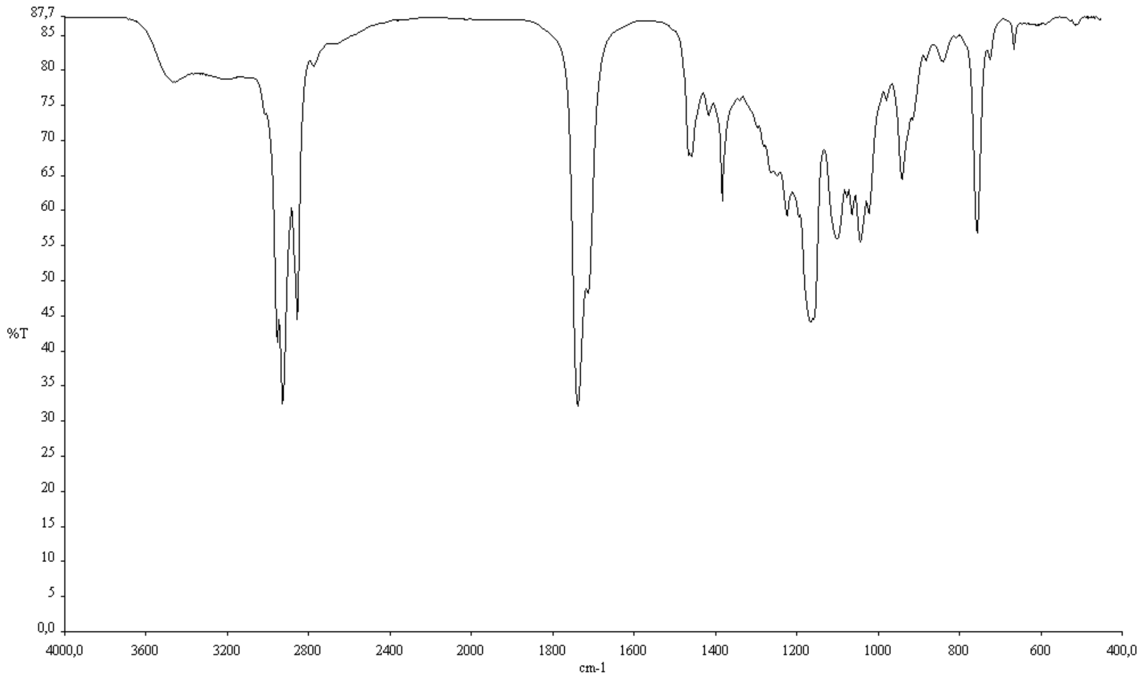

Figure S19. FT-IR spectrum of (1,3-dioxolan-4-yl)methyl octanate **5**: IR (wavenumber cm^{-1}; Transmittance %) 3558;77, 2930;32, 2858;44, 1739;32, 1492;68, 1384;61, 1166;44, 1045;55, 942;64,757;56.

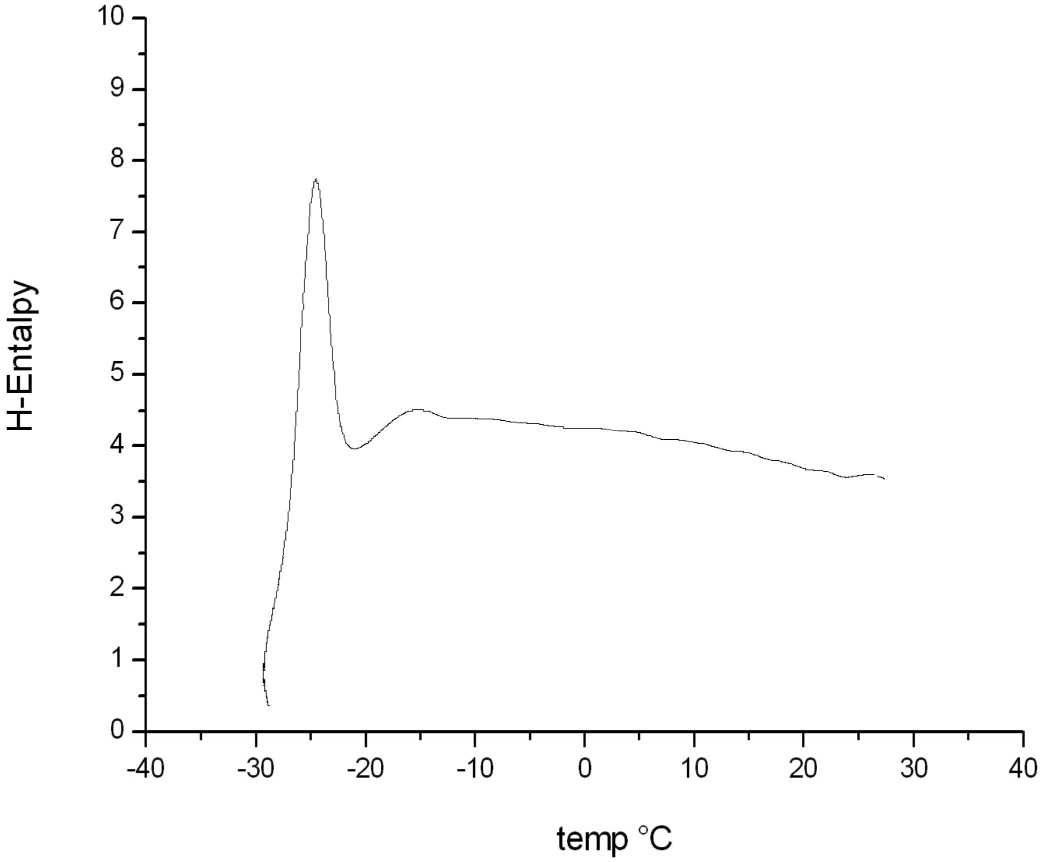

Figure S20. DSC (1,3-dioxolan-4-yl)methyl octanoate **5**.

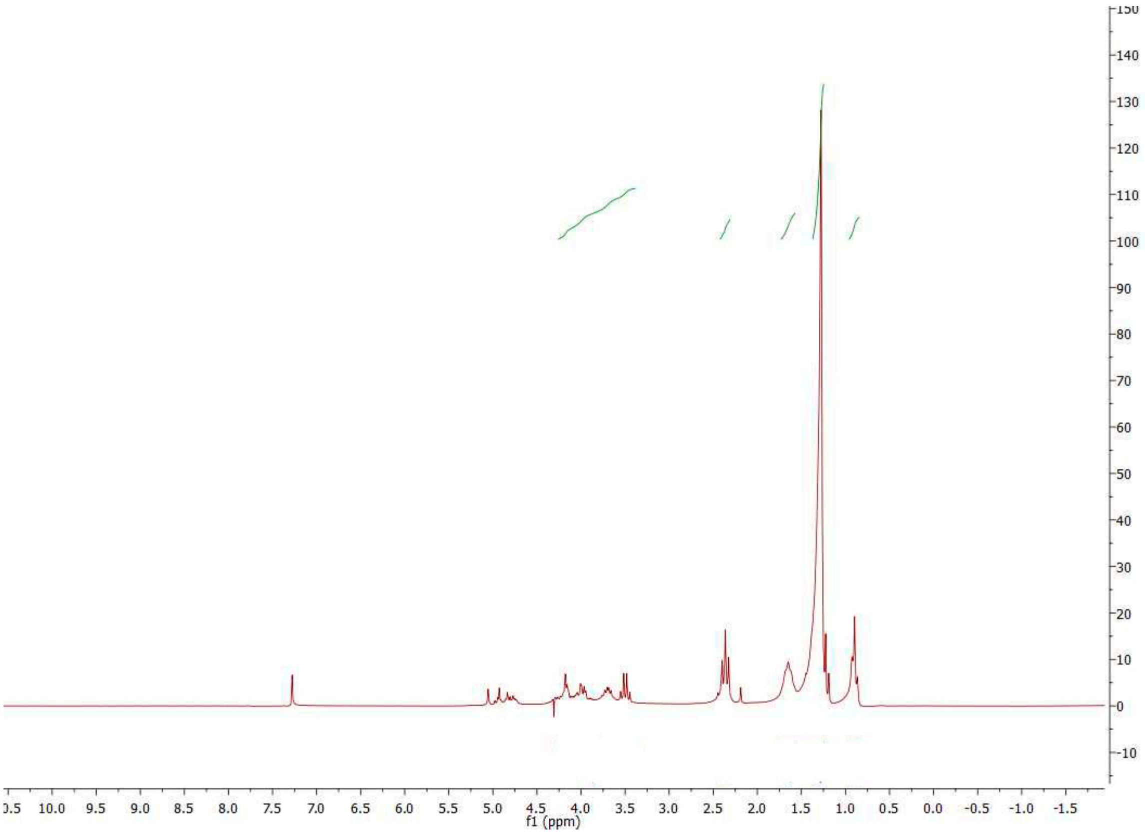

Figure S21. ^{1}H-NMR (200 MHz) spectrum of (1,3-dioxolan-4-yl)methyl dodecanoate **6**: ^{1}H-NMR (200 MHz) δ 5.14–3.82 (m, 6H), 3.71 (dq, *J* = 14.0, 5.0, 4.1 Hz, 2H), 2.37 (t, *J* = 7.6 Hz, 3H), 1.65 (t, *J* = 7.3 Hz, 3H), 1.42–1.22 (m, 9H), 0.95–0.82 (m, 3H).

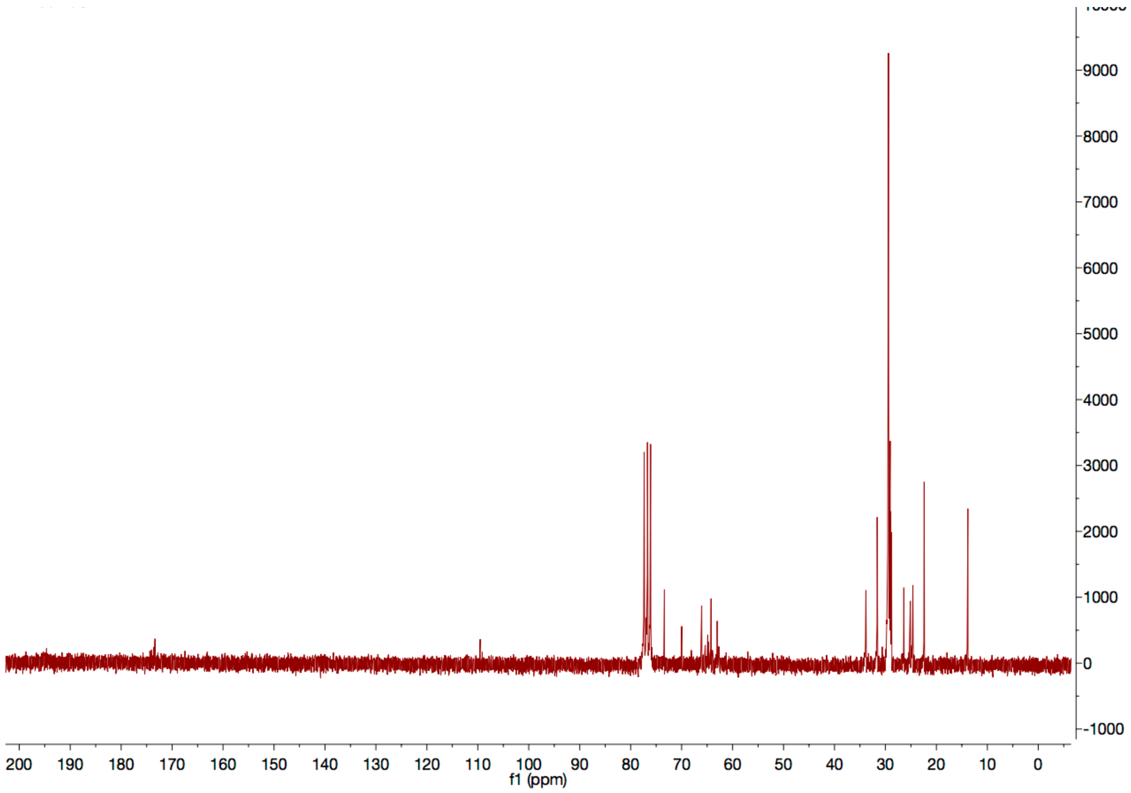

Figure S22. ^{13}C-NMR (200 MHz) spectrum of (1,3-dioxolan-4-yl)methyl dodecanoate **6**: ^{13}C-NMR (200 MHz) δ 178.71, 93.37, 77.35, 68.29, 63.59, 33.97, 29.30, 29.03, 28.94, 28.82, 28.77, 26.39, 24.60, 24.40, 22.38, 13.81**.**

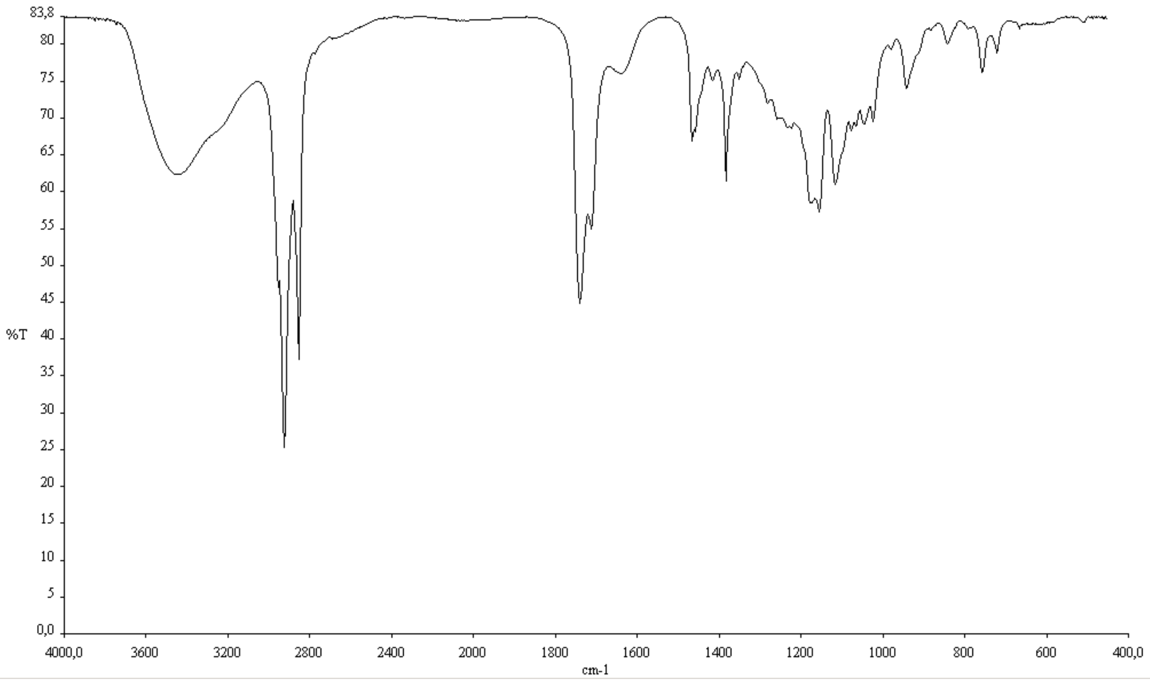

Figure S23. FT-IR spectrum of (1,3-dioxolan-4-yl)methyl dodecanoate **6**: IR (wavenumber cm^{-1}; Transmittance %) 3451;62, 2926;25, 2855;37, 1740;4 4, 1467;66, 1384;61, 1156;57, 1117;60, 956;74, 556;76.

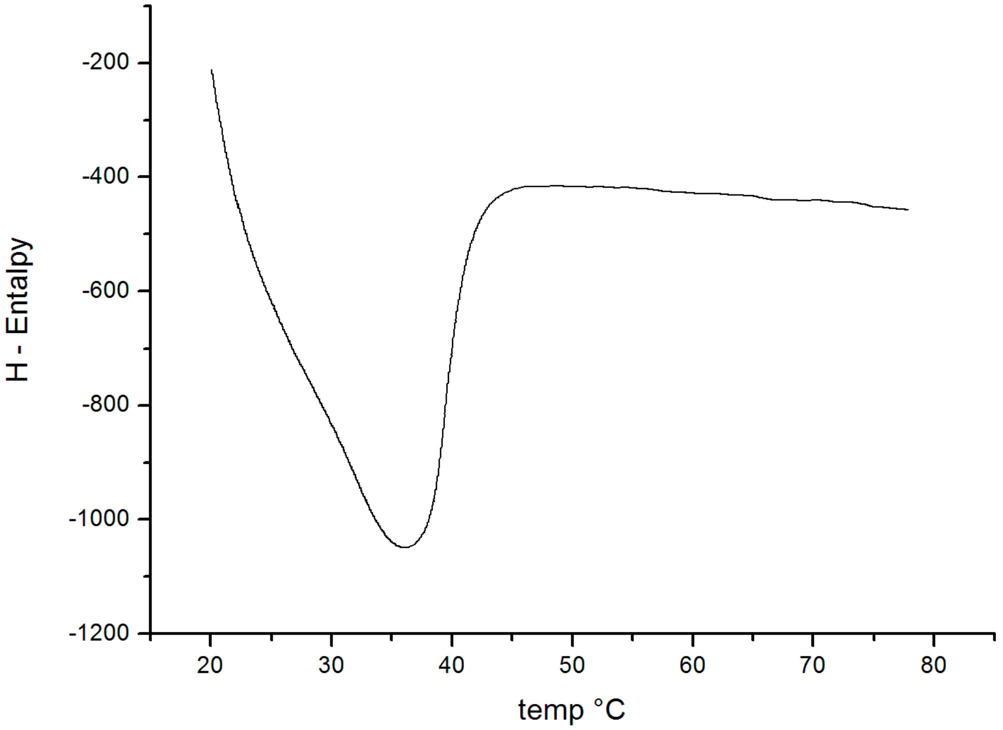

Figure S24. DSC of (1,3-dioxolan-4-yl)methyl dodecanoate **6**.

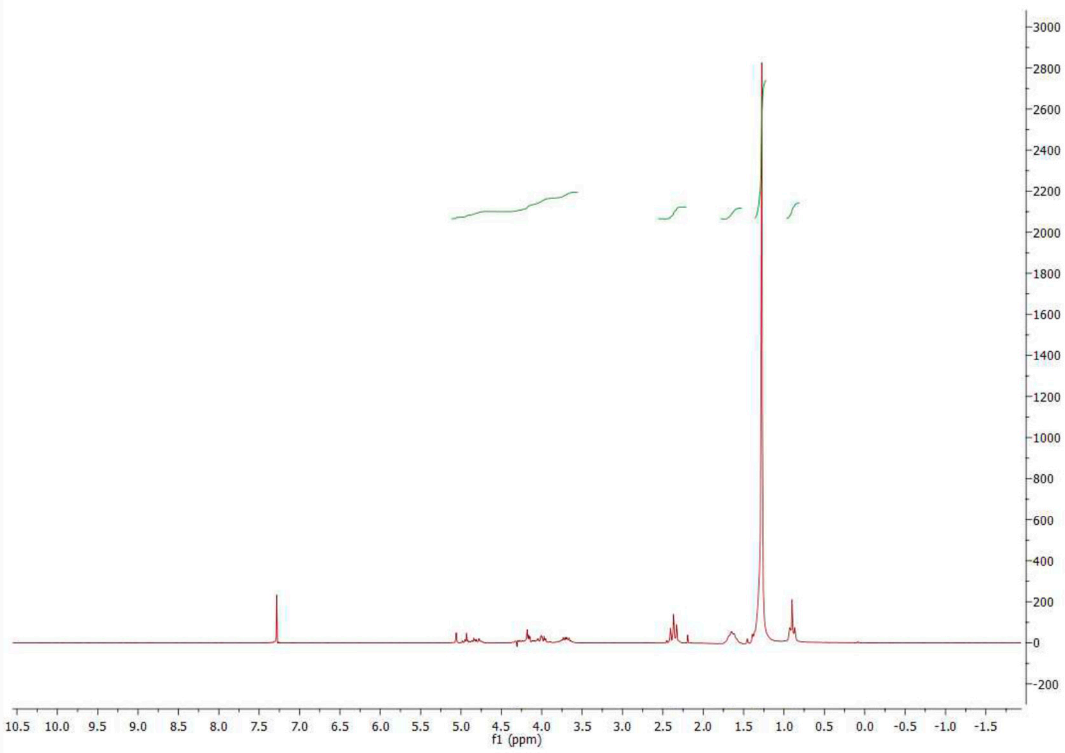

Figure S25. ^{1}H-NMR (200 MHz) spectrum of (1,3-dioxolan-4-yl)methyl palmitate **7**: ^{1}H-NMR (200 MHz) δ 5.06–3.52 (m, 6H), 2.35 (t, *J* = 7.5 Hz, 2H), 1.64 (t, *J* = 7.3 Hz, 3H), 1.26 (s, 27H), 0.89 (t, *J* = 6.3 Hz, 3H).

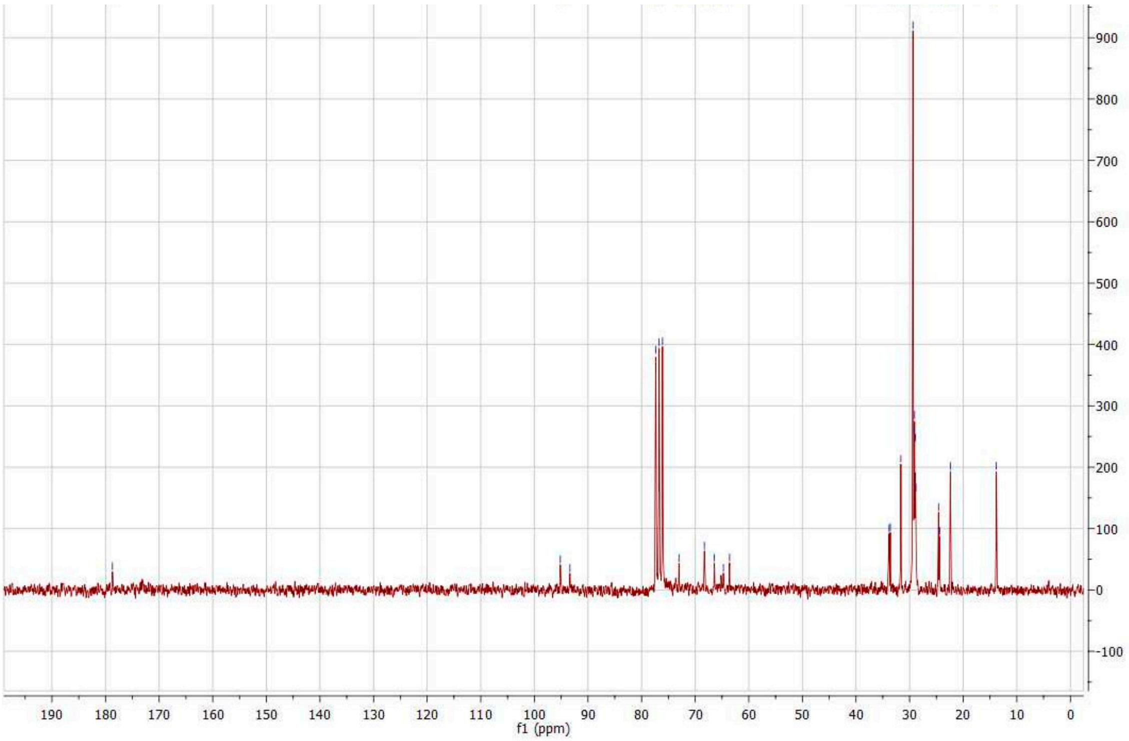

Figure S26. ^{13}C-NMR (200 MHz) spectrum of (1,3-dioxolan-4-yl)methyl palmitate **7**: ^{13}C-NMR (200 MHz) δ 178.71, 95.15, 77.36, 68.29, 66.43, 65.17, 33.97, 33.82, 33.59, 31.63, 29.39, 29.36, 29.30, 29.06, 28.95, 28.83, 28.77, 24.60, 24.40, 22.40, 13.82.

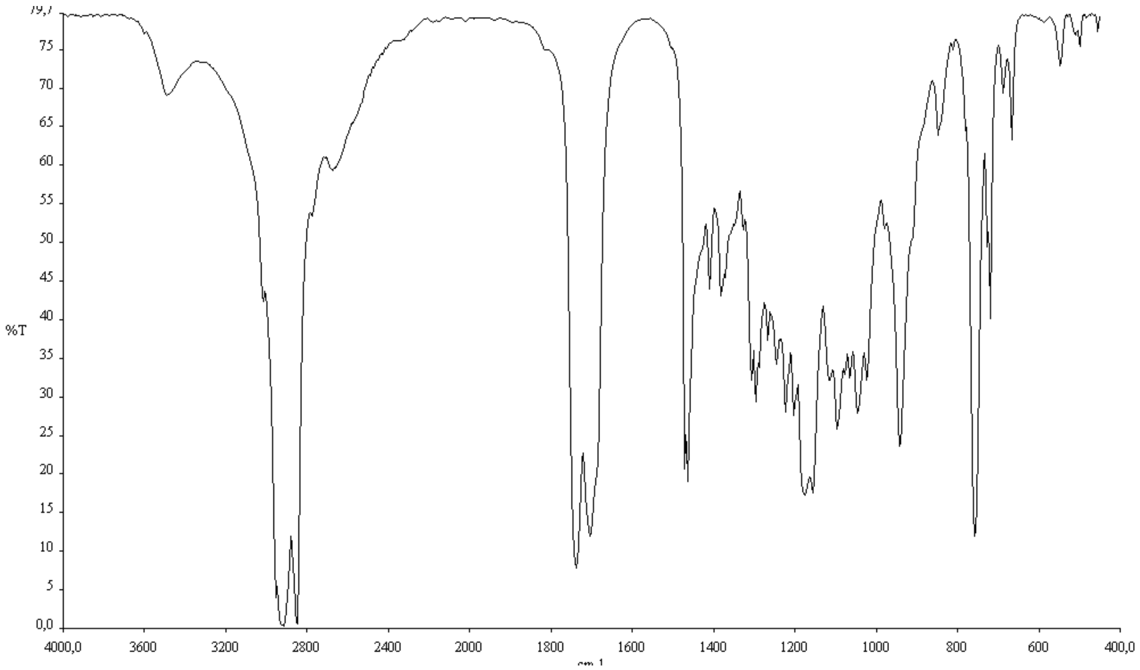

Figure S27. FT-IR spectrum of (1,3-dioxolan-4-yl)methyl palmitate **7**: IR (wavenumber cm^{-1}; Transmittance %) 2916;1, 2850;2, 1738;7, 1704;11, 1463;19, 1383;43, 1297;29, 1175;17, 1097;25, 1015;27, 943;23, 758;11, 720;40, 667;63.

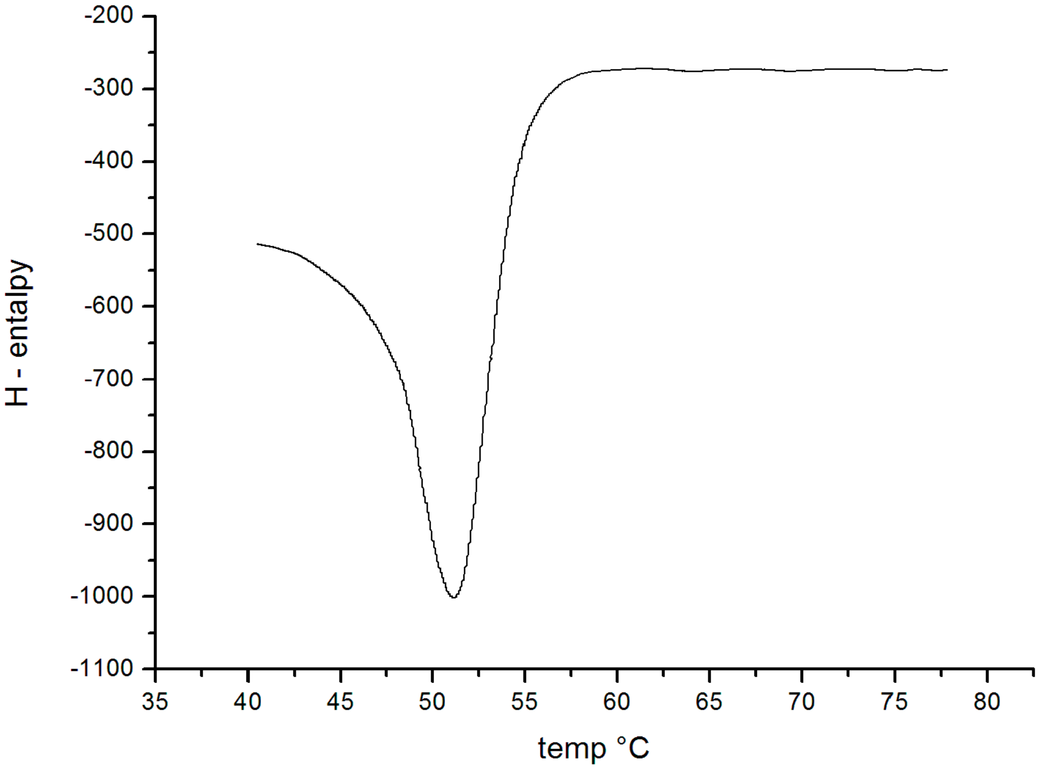

Figure S28. DSC of (1,3-dioxolan-4-yl)methyl palmitate **7.**

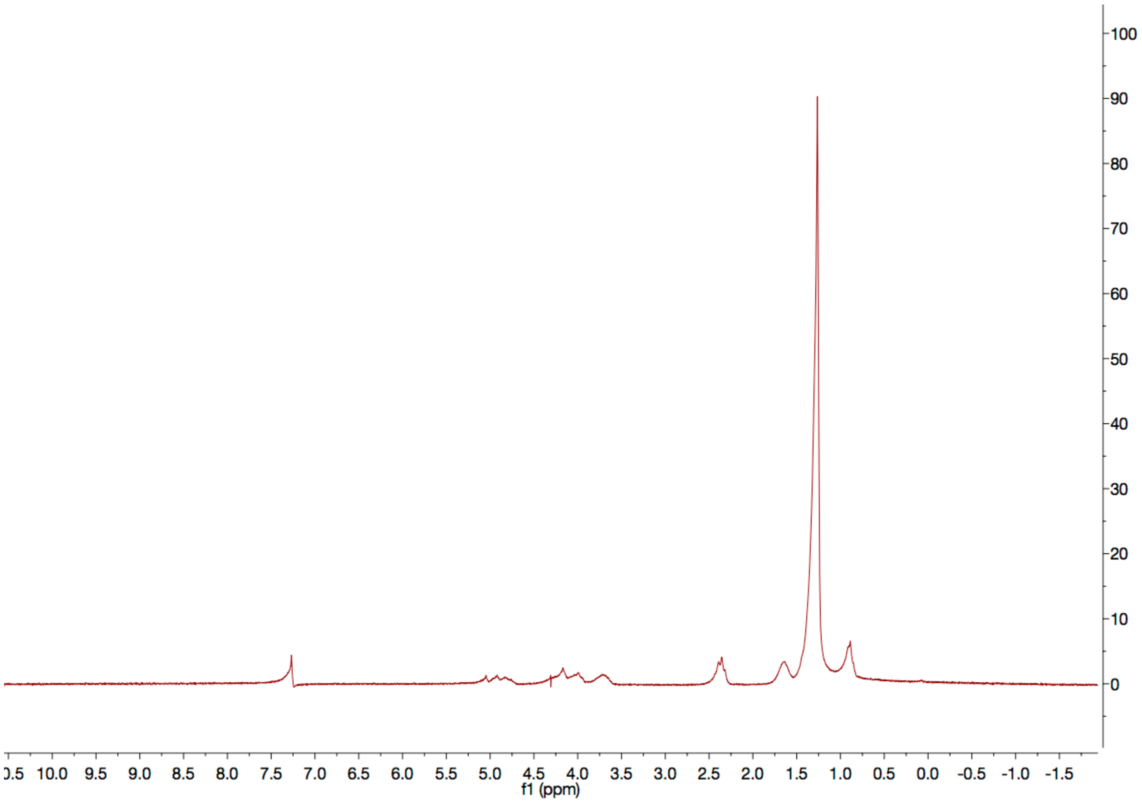

Figure S29. ^{1}H-NMR (200 MHz) spectrum of (1,3-dioxolan-4-yl)methyl stearate **8:** ^{1}H-NMR (200 MHz) δ 5.16–3.53 (m, 4H), 2.37 (s, 1H), 1.64 (s, 1H), 1.28 (d, J = 7.1 Hz, 25H), 0.97–0.82 (m, 3H).

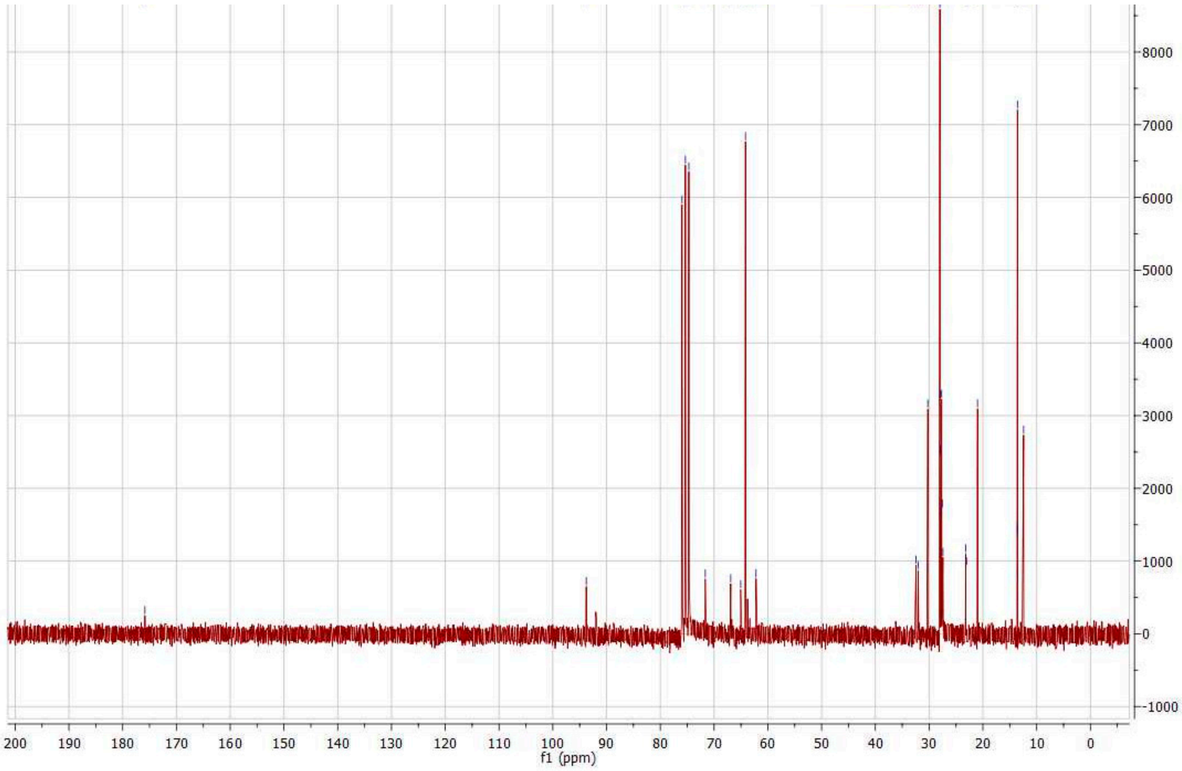

Figure S30. ^{13}C-NMR (200 MHz) spectrum of (1,3-dioxolan-4-yl)methyl stearate **8**: ^{13}C-NMR (200 MHz) δ 175.83, 93.76, 74.68, 71.63, 66.89, 65.04, 63.77, 62.18, 32.58, 32.42, 32.01, 28.01, 27.97, 27.91, 27.76, 27.56, 27.44, 27.39, 23.21, 23.05, 13.57, 12.42.

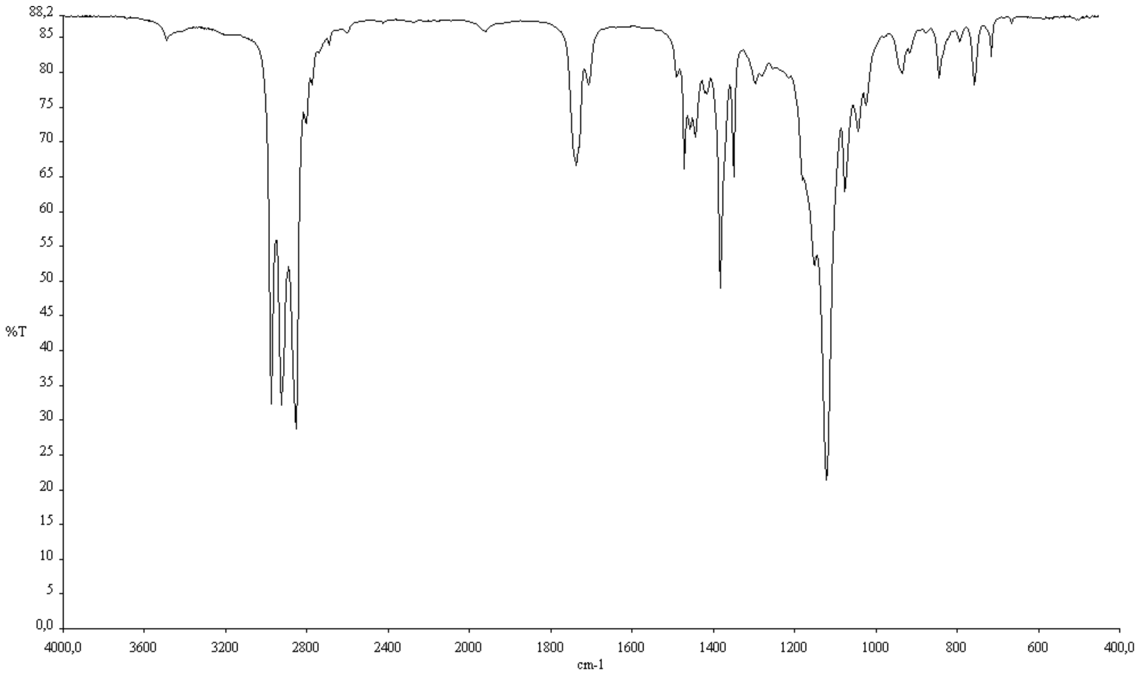

Figure S31. FT-IR spectrum of (1,3-dioxolan-4-yl)methyl stearate **8**: IR (wavenumber cm^{-1}; Transmittance %) 2977;32, 2927;32, 2855;28, 1737;66. 1472;66, 1383;49, 1350;65, 1122;21, 953;80, 830;79, 680;77.

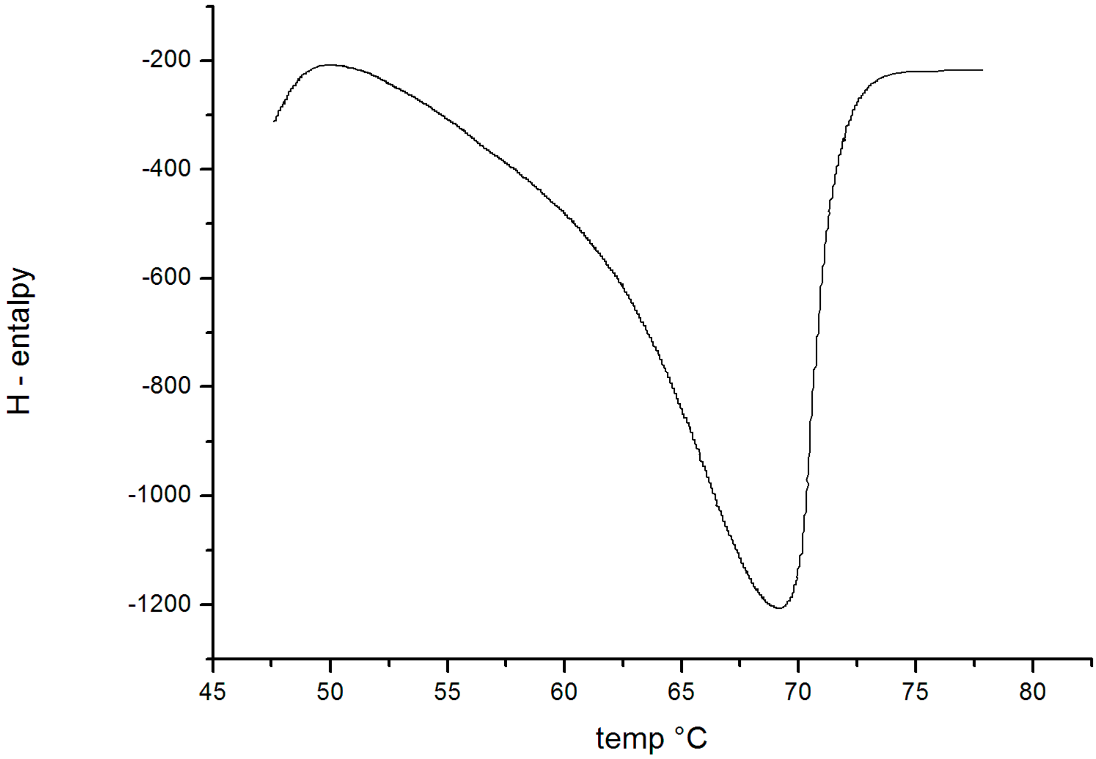

Figure S32. DSC of (1,3-dioxolan-4-yl)methyl stearate **8**.
